# The Program of Gene Transcription for a Single Differentiating Cell Type during Sporulation in Bacillus subtilis

**DOI:** 10.1371/journal.pbio.0020328

**Published:** 2004-09-21

**Authors:** Patrick Eichenberger, Masaya Fujita, Shane T Jensen, Erin M Conlon, David Z Rudner, Stephanie T Wang, Caitlin Ferguson, Koki Haga, Tsutomu Sato, Jun S Liu, Richard Losick

**Affiliations:** **1**Department of Molecular and Cellular Biology, Harvard UniversityCambridge, MassachusettsUnited States of America; **2**Department of Statistics, Harvard UniversityCambridge, MassachusettsUnited States of America; **3**International Environmental and Agricultural Science, Tokyo University of Agriculture and TechnologyFuchu, TokyoJapan

## Abstract

Asymmetric division during sporulation by Bacillus subtilis generates a mother cell that undergoes a 5-h program of differentiation. The program is governed by a hierarchical cascade consisting of the transcription factors: σ^E^, σ^K^, GerE, GerR, and SpoIIID. The program consists of the activation and repression of 383 genes. The σ^E^ factor turns on 262 genes, including those for GerR and SpoIIID. These DNA-binding proteins downregulate almost half of the genes in the σ^E^ regulon. In addition, SpoIIID turns on ten genes, including genes involved in the appearance of σ^K^_._ Next, σ^K^ activates 75 additional genes, including that for GerE. This DNA-binding protein, in turn, represses half of the genes that had been activated by σ^K^ while switching on a final set of 36 genes. Evidence is presented that repression and activation contribute to proper morphogenesis. The program of gene expression is driven forward by its hierarchical organization and by the repressive effects of the DNA-binding proteins. The logic of the program is that of a linked series of feed-forward loops, which generate successive pulses of gene transcription. Similar regulatory circuits could be a common feature of other systems of cellular differentiation.

## Abstract 2

A comprehensive genomic analysis of sporulation in *Bacillus subtilis* reveals a coordinated program of gene activation and repression, which involves 383 genes

## Introduction

A fundamental challenge in the field of development is to understand the entire program of gene expression for a single differentiating cell type in terms of an underlying regulatory circuit. This challenge can be met in part through recent advances in transcriptional profiling, which have made it possible to catalog changes in gene expression on a genome-wide basis (Brown and Botstein 1999). However, most systems of development involve multiple differentiating cell types, complicating the challenge of deciphering the program of gene expression for individual cell types. Also, many developmental systems are insufficiently accessible to genetic manipulation to allow genome-wide changes in gene expression to be understood in detail in terms of an underlying regulatory program. An understanding of how a cell differentiates from one type into another requires both a comprehensive description of changes in gene expression and an elucidation of the underlying regulatory circuit that drives the program of gene expression. Here we report our efforts to comprehensively catalog the program of gene expression in a primitive system of cellular differentiation, spore formation in the bacterium *Bacillus subtilis,* and to understand the logic of this program in terms of a simple regulatory circuit involving the ordered appearance of two RNA polymerase sigma factors and three positively and/or negatively acting DNA-binding proteins.

Spore formation in B. subtilis involves the formation of an asymmetrically positioned septum that divides the developing cell (sporangium) into unequal-sized progeny that have dissimilar programs of gene expression and distinct fates (Piggot and Coote 1976; Stragier and Losick 1996; Piggot and Losick 2002; Errington 2003). The two progeny cells are called the forespore (the smaller cell) and the mother cell. Initially, the forespore and the mother cell lie side by side, but later in development the forespore is wholly engulfed by the mother cell, pinching it off as a cell within a cell. The forespore is a germ cell in that it ultimately becomes the spore and, upon germination, gives rise to vegetatively growing cells. The mother cell, on the other hand, is a terminally differentiating cell type that nurtures the developing spore but eventually undergoes lysis to liberate the fully ripened spore when morphogenesis is complete. The entire process of spore formation takes 7–8 h to complete with approximately 5 h of development taking place after the sporangium has been divided into forespore and mother-cell compartments.

Much is known about the transcription factors that drive the process of spore formation, and in several cases transcriptional profiling has been carried out to catalog genes switched on or switched off by individual sporulation regulatory proteins (Fawcett et al. 2000; Britton et al. 2002; Eichenberger et al. 2003; Feucht et al. 2003; Molle et al. 2003a). Here we have attempted to go a step further by comprehensively elucidating the program of gene expression for a single cell type in the developing sporangium. For this purpose we focused on the mother cell and its 5-h program of gene expression. Gene expression in the mother cell is governed by five positively and/or negatively acting transcription factors. These are the sigma factors σ^E^ and σ^K^ and the DNA-binding proteins GerE, GerR (newly characterized in the present study), and SpoIIID.

**Figure 1:**
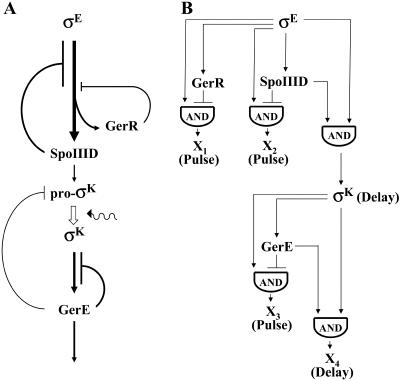
The Mother-Cell Line of Gene Transcription (A) Gene transcription is governed by a hierarchical regulatory cascade that involves gene activation and gene repression. The σ^E^ factor turns on a large regulon that includes the genes for GerR and SpoIIID. These DNA-binding proteins, in turn, block further transcription of many of the genes that had been activated by σ^E^. SpoIIID is also an activator, and it turns on genes required for the appearance of pro-σ^K^. The conversion of pro-σ^K^ to mature σ^K^ is governed by a signal emanating from the forespore as represented by the squiggle. Next, σ^K^ activates the subsequent regulon in the cascade, which includes the gene for the DNA-binding protein GerE. Finally, GerE, which, like SpoIIID, is both an activator and a repressor, turns on the final regulon in the cascade while also repressing many of the genes that had been activated by σ^K^. The thickness of lines represents the relative abundance of genes activated (arrows) or repressed (lines ending in bars) by the indicated regulatory proteins. (B) The regulatory circuit is composed of two coherent FFLs linked in series and three incoherent FFLs. In the first coherent FFL, σ^E^ turns on the synthesis of SpoIIID, and both factors act together to switch on target genes, including genes involved in the appearance of σ^K^. Likewise, in the second coherent FFL, σ^K^ directs the synthesis of GerE, and the two factors then act together to switch on target genes (X_4_). The σ^E^ factor and SpoIIID also constitute an incoherent FFL in which SpoIIID acts as a repressor to downregulate the transcription of a subset of the genes (X_2_) that had been turned on by σ^E^. Similar incoherent FFLs are created by the actions of σ^E^ and GerR (X_1_) and by σ^K^ and GerE (X_3_), with GerR and GerE repressing genes that had been switched on by σ^E^ and σ^K^, respectively. The AND symbols indicate that the FFLs operate by the logic of an AND gate in that the output (either gene activation or a pulse of gene expression) requires the action of both transcription factors in the FFL (see Mangan and Alon 2003). For example, σ^K^ and GerE are both required for the activation of X_4_ genes, whose induction is delayed compared to genes that are turned on by σ^K^ alone. Similarly, both σ^E^ and the delayed appearance of GerR are anticipated to create a pulse of transcription of X_1_ genes.

The appearance of these regulatory proteins is governed by a hierarchical regulatory cascade of the form: σ^E^→SpoIIID/GerR→σ^K^→GerE (Figure 1A) in which σ^E^ is the earliest-acting factor specific to the mother-cell line of gene expression (Zheng and Losick 1990; results presented herein). The σ^E^ factor is derived from an inactive proprotein, pro-σ^E^ (LaBell et al. 1987), whose synthesis commences before asymmetric division (Satola et al. 1992; Baldus et al. 1994), but whose continued synthesis becomes strongly biased to the mother cell after asymmetric division (Fujita and Losick 2002 2003). Proteolytic conversion to mature σ^E^ takes place just after asymmetric division (Stragier et al. 1988) and is triggered by an intercellular signal transduction pathway involving a secreted signaling protein that is produced in the forespore under the control of the forespore-specific transcription factor σ^F^ (Hofmeister et al. 1995; Karow et al. 1995; Londono-Vallejo and Stragier 1995). Transcriptional profiling has established that σ^E^ turns on an unusually large regulon consisting of 262 genes, which are organized in 163 transcription units (Eichenberger et al. 2003; results presented herein). Among the targets of σ^E^ are the genes for the DNA-binding proteins SpoIIID and GerR (Kunkel et al. 1989; Stevens and Errington 1990; Tatti et al. 1991; Wu and Errington 2000; results presented herein). SpoIIID is both a negatively acting protein that switches off the transcription of certain genes that have been activated by σ^E^ and a positively acting protein that acts in conjunction with σ^E^-containing RNA polymerase to switch on additional genes, including genes involved in the appearance of σ^K^ (Kroos et al. 1989).

The appearance of σ^K^ is a critical control point that involves multiple levels of regulation: transcription, DNA recombination, and proprotein processing. SpoIIID both activates the transcription of the 5′ coding region for σ^K^ *(spoIVCB)* and that for a site-specific DNA recombinase *(spoIVCA)* (Kunkel et al. 1990; Halberg and Kroos 1994) that joins the 5′ coding sequence to the 3′ coding region by the excision of an intervening sequence of 48 kb called *skin* (Stragier et al. 1989). Finally, the product of the intact coding sequence is an inactive proprotein, pro-σ^K^ (Kroos et al. 1989), whose conversion to mature σ^K^ (as in the case of pro-σ^E^) is governed by a complex, intercellular signal transduction pathway involving a secreted signaling protein that is produced in the forespore under the control of the forespore-specific transcription factor σ^G^ (Cutting et al. 1990, 1991a; Lu et al. 1990). The signal transduction pathway helps to coordinate the appearance of σ^K^ in the mother cell with the timing of events taking place in the forespore. The σ^K^ factor turns on an additional gene set that includes the gene for GerE (Cutting et al. 1989), a DNA-binding protein that is responsible for activating the final temporal class of genes in the mother-cell line of gene expression (Zheng et al. 1992).

Other than the case of σ^E^, little was previously known about the full set of genes, whose transcription is governed by the five regulators in the mother-cell line of gene expression—indeed, nothing at all in the case of GerR, whose function had previously been uncharacterized. Here we present evidence indicating that the program of mother-cell-specific gene transcription involves the activation of at least 383 genes (242 transcription units), representing 9% of the genes in the B. subtilis genome. We explain the pattern of transcription of each of these genes in terms of the action of the five regulatory proteins that govern the mother-cell program of gene transcription. Our results reveal that the program chiefly consists of a series of pulses in which large numbers of genes are turned on and are then turned off shortly thereafter by the action of the next regulatory protein in the hierarchy. Evidence is also presented that this repression is critical for proper morphogenesis. Finally, we show that the mother-cell program of gene transcription can be understood in terms of a simple regulatory circuit involving a linked series of feed-forward loops (FFLs) that are responsible for generating pulses of gene transcription. We propose that this regulatory circuit will serve as a model for understanding other programs of cellular differentiation.

## Results

### Transcriptional Profiling

Our strategy for elucidating the mother-cell program of gene transcription was to carry out transcriptional profiling at hourly intervals during sporulation at 37 °C, starting just after asymmetric division and ending before the time at which lysis of the mother cell had commenced. At each time point, RNA from cells mutant for the transcriptional regulator that was maximally active at that time interval was compared against RNA from cells mutant for the next transcription factor in the hierarchy or, in the case of the last regulatory protein in the hierarchy, GerE, against RNA from wild-type cells. Thus, at hour 2.5, RNA from cells mutant for σ^E^ (strain PE437) was compared against RNA from cells (strain PE436) that were wild type for σ^E^ but mutant for the next regulatory protein in the sequence, SpoIIID. Likewise, at hour 3.5, RNA from cells that were mutant for SpoIIID (strain PE456) was compared against RNA from cells that were mutant for σ^K^ (strain PE452). (Strains PE456 and PE452 were additionally mutant for σ^G^ to eliminate indirect effects of the presence or absence of SpoIIID on the activity of the forespore-specific transcription factor. Although SpoIIID has no direct effect on σ^G^, the absence of negative feedback on several σ^E^-controlled genes [see below] in the strain mutated for *spoIIID* could have had indirect consequences on σ^G^ activity.) Likewise, at hour 4.5, RNA from cells that were mutant for σ^K^ (strain PE455) was compared against RNA from cells mutant for GerE (strain PE454). Finally, at hours 5.5 and 6.5, RNA from cells mutant for GerE was compared against RNA from wild-type cells (PY79). Three transcriptional-profiling analyses were carried out for each of these time points, using three independent preparations of RNA from each of the two cultures of cells that were being compared against each other. The complete dataset for these experiments is presented in Table S1, and transcriptional profiles for representative genes are displayed in Table 1.

**Table 1:**
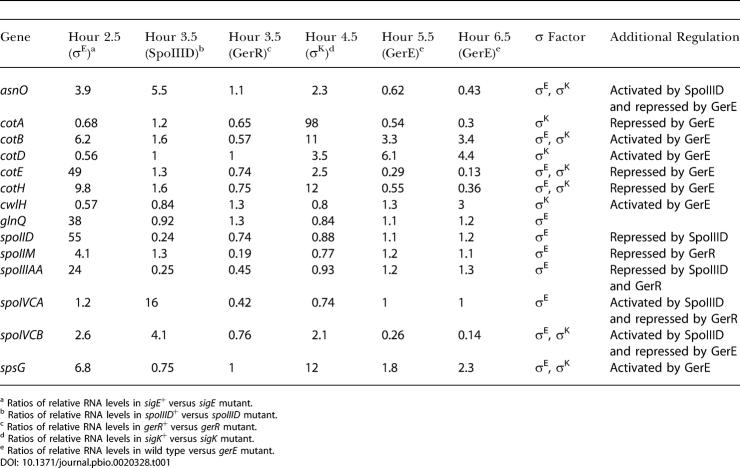
Transcriptional Profile of Representative Genes

^a^ Ratios of relative RNA levels in *sigE* ^+^ versus *sigE* mutant

^b^ Ratios of relative RNA levels in *spoIIID* ^+^ versus *spoIIID* mutant

^c^ Ratios of relative RNA levels in *gerR* ^+^ versus *gerR* mutant

^d^ Ratios of relative RNA levels in *sigK* ^+^ versus *sigK* mutant

^e^ Ratios of relative RNA levels in wild type versus *gerE* mutant

In addition to the four previously known members of the hierarchical regulatory cascade, one of the genes in the σ^E^ regulon is inferred to encode a previously uncharacterized DNA-binding protein YlbO (Wu and Errington 2000; Eichenberger et al. 2003). Additional transcriptional-profiling experiments were carried out to assess the function of this putative regulatory protein.

### Updating the σ^E^ Regulon

We previously reported that the σ^E^ regulon is composed of 253 genes, organized in 157 transcription units. Since then two additional σ^E^-controlled genes, *yjcA* (Kuwana et al. 2003) and *ctpB (yvjB)* (Pan et al. 2003), have been identified. These genes were found to be transcribed in a σ^E^-dependent manner during sporulation in our previous analysis, but they were not significantly induced in cells engineered to produce σ^E^ during growth and hence had not been included in our original list of σ^E^-controlled genes. In addition, results presented here (see below) show that one gene, *ypqA,* and two operons, *yhcOP* and *yitCD,* that are chiefly under the control of σ^K^, are also transcribed, albeit at a low level, in a σ^E^-dependent manner. These and other considerations (see below) bring the current total number of genes in the σ^E^ regulon to 262 and the total number of transcription units to 163 (Table 2).

**Table 2:**
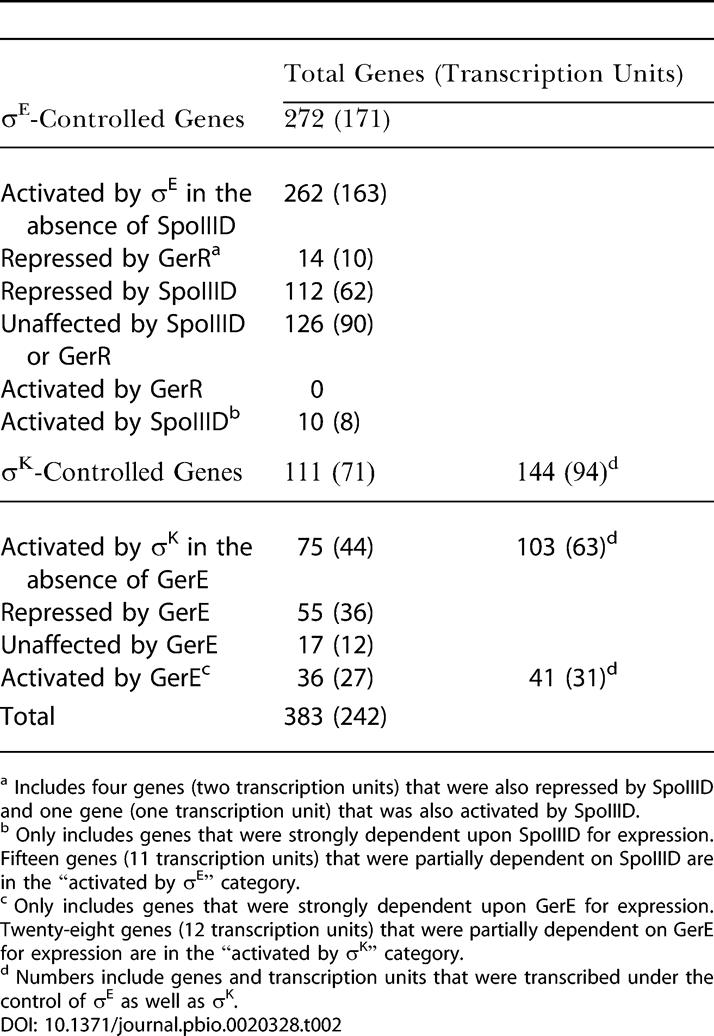
Genes Activated in the Mother-Cell Line of Gene Expression

^a^ Includes four genes (two transcription units) that were also repressed by SpoIIID and one gene (one transcription unit) that was also activated by SpoIIID

^b^ Only includes genes that were strongly dependent upon SpoIIID for expression. Fifteen genes (11 transcription units) that were partially dependent on SpoIIID are in the “activated by σ^E^” category

^c^ Only includes genes that were strongly dependent upon GerE for expression. Twenty-eight genes (12 transcription units) that were partially dependent on GerE for expression are in the “activated by σ^K^” category

^d^ Numbers include genes and transcription units that were transcribed under the control of σ^E^ as well as σ^K^

This updated description of the σ^E^ regulon does not include genes and transcription units that are additionally strongly dependent upon SpoIIID for their transcription because our previous transcriptional-profiling experiments were performed with a strain that was mutant for SpoIIID. SpoIIID is a DNA-binding protein that acts in conjunction with σ^E^-containing RNA polymerase (Kroos et al. 1989; Kunkel et al. 1989; Halberg and Kroos 1994). Therefore, as a starting point for the present study, we investigated the influence of SpoIIID on the global pattern of σ^E^-directed transcription. As we shall see, this analysis revealed ten genes (representing eight transcription units) that were strongly dependent upon SpoIIID for expression and were not expressed under the control of σ^E^ alone**,** bringing the present total number of genes in the σ^E^ regulon to 272 and the total number of transcription units to 171 (Table 2).

### SpoIIID Is Both a Repressor and an Activator of Genes Whose Transcription Is Dependent Upon σ^E^

**Figure 2:**
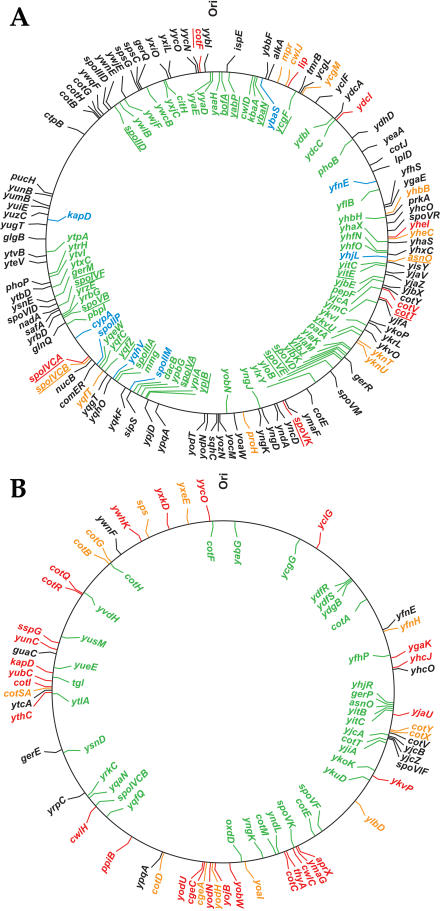
Location of Genes in the σ^E^ and σ^K^ Regulons and Their Regulation by DNA-Binding Proteins (A) The σ^E^ regulon and its modulation by SpoIIID and GerR. The first gene of each σ^E^-controlled transcription unit identified by transcriptional profiling is indicated. In the inner circle, genes repressed by SpoIIID are green, and genes repressed by GerR are blue. In the outer circle, genes partially dependent on SpoIIID for expression are orange, and genes strongly dependent on SpoIIID are red. Underlined are SpoIIID-controlled genes for which SpoIIID binding to their upstream sequences has been demonstrated biochemically. Genes unaffected by SpoIIID or GerR are indicated in black. (B) The σ^K^ regulon and its modulation by GerE. The first gene of each σ^K^-controlled transcription unit identified by transcriptional profiling is indicated. In the inner circle, genes repressed by GerE are green. In the outer circle, genes partially dependent on GerE for expression are orange, and genes strongly dependent on GerE are red. Genes unaffected by GerE are indicated in black.

Transcriptional profiling revealed that SpoIIID had profound effects on the global pattern of σ^E^-directed gene transcription. As many as 181 genes were found to be downregulated in the presence of SpoIIID. Of these, 148 had previously been identified as being activated in a σ^E^-dependent manner, at least 112 of which (representing 62 transcription units) were bona fide members of the σ^E^ regulon (that is, they met multiple criteria for being under the direct control of σ^E^) (see Table S2). Therefore, a principal function of SpoIIID is to inhibit the transcription of a substantial proportion (greater than 40%) of the genes whose transcription had been activated by σ^E^ prior to the appearance of SpoIIID. Members of the σ^E^ regulon that are downregulated by SpoIIID are colored green in Figure 2A.

SpoIIID not only repressed many genes in the σ^E^ regulon but also stimulated or activated the transcription of many others. At least 70 genes were identified whose transcription was upregulated by SpoIIID (Table S2), but in many cases these genes were not members of the σ^E^ regulon, and the effect of SpoIIID could have been indirect. Examples are seven genes *(cysK , cysH, cysP, sat, cysC, yoaD,* and *yoaB)* from the S-box regulon (Grundy and Henkin 1998) and two genes *(argC* and *argJ)* from the arginine biosynthesis operon (Smith et al. 1989). In other cases, however, SpoIIID stimulated or activated the transcription of genes that had been reported to be under the control of σ^E^. Thus, 13 *(asnO, cwlJ, proH, proJ, spoIVCA, spoIVCB, spoVK , yhbB, yheC, yheD, yknT, yknU,* and *yknV)* of the genes whose transcription was upregulated by SpoIIID had previously been assigned to the σ^E^ regulon, and four others *(mpr, ycgM, ycgN,* and *yqfT)* were known to be under σ^E^ control but had not met all of the criteria for assignment to the σ^E^ regulon (Eichenberger et al. 2003). In two of these 17 cases *(spoIVCA* and *spoVK),* the dependence on SpoIIID was almost complete, whereas in the other 15 the dependence was partial.

Our analysis revealed eight additional genes *(cotF, cotT, cotV, cotW, lip, ydcI, yheI,* and *yheH)* that were almost completely dependent on SpoIIID for their transcription and that are likely to be under the dual control of σ^E^ and SpoIIID. Thus, in addition to repressing at least 112 members of the σ^E^ regulon, SpoIIID activates the transcription of 25 other members of the regulon, representing 19 transcription units. The 15 σ^E^-transcribed genes (11 transcription units) whose expression was partially dependent upon SpoIIID are indicated in orange in Figure 2B, and those whose expression was completely dependent on the DNA-binding protein are indicated in red (ten genes; eight transcription units).

Evidently, then, SpoIIID plays a pivotal role in the mother-cell line of gene expression, negatively or positively affecting the transcription of many members of the σ^E^ regulon. It was therefore important to determine whether the genes so affected were direct targets of the DNA-binding protein. For this purpose, we used three complementary approaches to identifying binding sites for SpoIIID: biochemical analysis by gel electrophoretic mobility-shift assays (EMSAs) and DNAase I footprinting, in vivo analysis by chromatin-immunoprecipitation in combination with gene microarrays (ChIP-on-chip), and the identification of SpoIIID-binding sequences by computational analysis.

### Biochemical Identification of SpoIIID-Binding Sites

**Figure 3:**
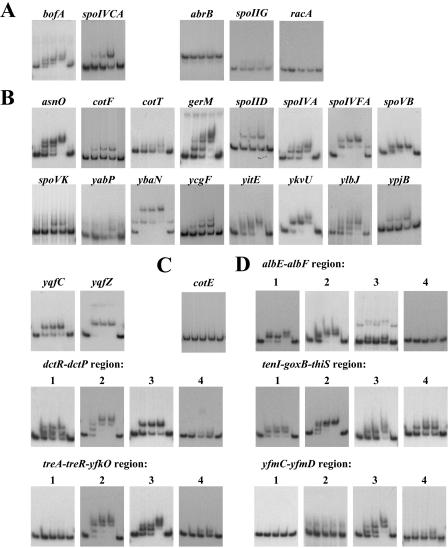
Gel Electrophoretic Mobility-Shift Analysis of SpoIIID Binding DNA fragments of interest were amplified by PCR, gel-purified, and end-labeled using [γ-^32^P]-ATP and polynucleotide kinase. Purified SpoIIID was added at increasing concentrations (0 nM for lanes 1 and 5, 50 nM for lane 2, 100 nM for lane 3, and 200 nM for lane 4) and incubated at room temperature for 30 min before loading on to a nondenaturing gel containing 6% polyacrylamide. With the exception of (D), the DNA fragments corresponded to the upstream regions of the indicated genes. See Materials and Methods for the identity (coordinates) of the specific DNA sequences used in the analyses. (A) Gel shifts for known targets of SpoIIID (*bofA* and *spoIVCA*), representing positive controls, and genes (*abrB, spoIIGA,* and *racA*) under the control of another DNA-binding protein (Spo0A), representing negative controls. (B) Gel shifts for genes identified as possible targets of SpoIIID by transcriptional profiling. (C) Gel shift for *cotE*. Expression of *cotE* from its P2 promoter is strongly dependent on SpoIIID. No binding of SpoIIID to the upstream sequence for *cotE* is observed, suggesting that the effect of SpoIIID on transcription from the P2 promoter is indirect. (D) Gel shifts for chromosomal regions strongly enriched for SpoIIID binding as judged by ChIP-on-chip analysis. For each region, four consecutive DNA fragments of approximately 400 nucleotides in length were analyzed.

We selected 18 of the newly identified SpoIIID-regulated genes for EMSA analysis, mostly on the basis of the importance of their role in sporulation. As positive controls, we subjected two previously known targets of SpoIIID, *bofA* and *spoIVCA* (Halberg and Kroos 1994), to EMSA analysis, and as negative controls three Spo0A-regulated genes (Molle et al. 2003a), *abrB, racA,* and *spoIIGA* (Figure 3A). SpoIIID exhibited binding to the upstream sequence of all 18 of the selected genes (Figure 3B). In some cases (those of *asnO, gerM, spoIVA, spoIVFA, ybaN, ycgF, yitE, ykvU,* and *ylbJ*) additional shifted bands were detected at high concentrations of SpoIIID, which may indicate the presence of two or more SpoIIID-binding sites with distinct binding affinities.

In addition, we also subjected the upstream region of *cotE* to EMSA analysis (Figure 3C). The *cotE* gene is transcribed from two promoters: a σ^E^-controlled promoter called P1 and a second promoter called P2 that strongly depends on SpoIIID (Zheng and Losick 1990). It had been assumed that transcription from P2 is under the dual control of σ^E^ and SpoIIID, but EMSA analysis failed to reveal a binding site for SpoIIID, and other work presented below indicates that transcription from *cotE* P2 is governed by σ^K^ rather than by σ^E^. We conclude that the SpoIIID dependence of *cotE* P2 is an indirect consequence of the dependence of σ^K^ synthesis on SpoIIID.

To obtain further evidence for direct interaction by SpoIIID and to investigate the mechanism by which SpoIIID inhibits transcription, we subjected the promoter regions of three genes *(spoIID, spoIIIAA,* and *spoVE)* identified as being under the negative control of SpoIIID to DNAase I footprinting analysis. SpoIIID protected two regions in the upstream sequence of *spoIID* from DNAase I digestion (Figure S1). One region (extending from positions −10 to −28 on the top strand and from −18 to −35 on the bottom strand) overlapped with the −10 element of the σ^E^ promoter, and the other (extending from −33 to −52 on the top strand) overlapped with the −35 element. The binding site for SpoIIID also overlapped with the promoter in the case of *spoIIIAA,* in this case protecting a single sequence that included the −35 element (extending from −21 to −45 on the top strand and from −30 to −48 on the bottom strand). Finally, the regulatory sequence of *spoVE* exhibited two binding sites, one (extending from +16 to −1 on the bottom strand) that was located in the vicinity of the predominant σ^E^-controlled promoter (P2) for this gene and another further upstream, overlapping with a secondary promoter (P1) (extending from +13 to −7 on the top strand). Thus, repression of the promoters of *spoIID, spoIIIAA,* and *spoVE* by SpoIIID is likely to be a direct consequence of the binding of the sporulation regulatory protein to the promoter in such a way as to compete with binding by σ^E^–RNA polymerase.

### SpoIIID Binds to Some Sites that Do Not Correspond to Genes under Its Control

ChIP-on-chip analysis was carried out as described in Materials and Methods and previously (Molle et al. 2003a, 2003b), using DNA–protein complexes from formaldehyde-treated cells at hour 3 of sporulation. After sonication, SpoIIID–DNA complexes were precipitated with antibodies against SpoIIID. Next, after reversal of the cross-links, the precipitated DNAs were amplified by PCR in the presence of cyanine 5-dUTP. In parallel, total sonicated DNA from the formaldehyde-treated cells (i.e., DNA that had not been subjected to immunoprecipitation) was similarly amplified, but in the presence of cyanine 3-dUTP. The two differentially labeled DNAs were combined and hybridized to the same batch of DNA microarrays that were used for the transcriptional-profiling experiments. Transcriptional profiling was carried out with three independent preparations of formaldehyde-treated cells, twice with two of the preparations and once with the third, for a total of five analyses. An enrichment factor was calculated for each gene, representing the enrichment of that gene by immunoprecipitation relative to DNA that had not been subjected to immunoprecipitation, and the entire dataset is displayed in Table S3.

Thirty-one genes, corresponding to 26 regions of the chromosome, were found to be enriched by immunoprecipitation by a factor of two or greater. Only seven of the regions *(cotF, lip, spoIIIAF, spoVD, ycgF, yhbH,* and *ykvI)* identified by the ChIP-on-chip analysis were in close proximity to a gene that was differentially expressed in the SpoIIID transcriptional-profiling experiments. Thus, in only a small number of cases did ChIP-on-chip analysis support the idea that a gene under SpoIIID control was a direct target of the DNA-binding protein. Our interpretation of these findings is that ChIP-on-chip is less sensitive for detecting SpoIIID-binding sites than it is for the B. subtilis DNA-binding proteins CodY (Molle et al. 2003b), Spo0A (Molle et al. 2003a), and RacA (Ben-Yehuda et al. 2003). Likely contributing to this decreased sensitivity is the fact that SpoIIID is present in only one of the two chromosome-containing compartments (the mother cell) of the sporangium and that its concentration is low (∼1 μM; Zhang et al. 1997).

While providing support for only a small proportion of the herein identified targets of SpoIIID regulation, ChIP-on-chip analysis, nonetheless, proved to be revealing. Specifically, we found that SpoIIID bound to many regions of the chromosome that did not correspond to genes under its negative or positive control. Were these regions bona fide SpoIIID-binding sites? To address this question, we subjected five regions that were most enriched for SpoIIID-binding *(albE*–*albF, dctR*–*dctP, tenI*–*goxB*–*thiS, treA*–*treR–yfkO,* and *yfmC–yfmD)* to EMSA analysis (Figure 3D). Given that SpoIIID was not exerting a transcriptional effect in these regions, we reasoned that the sites to which SpoIIID was binding might not reside in upstream regulatory regions and could instead be located in coding sequences. We therefore scanned across each of the five chromosomal regions by EMSA using successive DNA fragments of about 400 bp in length. The results showed that each of the five regions contained more than one binding site for SpoIIID and that some of these binding sites were indeed located within protein-coding sequences. (The presence of more than one binding site in each region may have facilitated their detection by the ChIP-on-chip analysis.) We conclude that SpoIIID binds to some sites on the chromosome at which it does not function as a transcriptional regulator. Conceivably, it plays an architectural role in the folding of the chromosome in the mother cell in addition to its role as a transcriptional regulator. Moqtaderi and Struhl (2004) have similarly found that in Saccharomyces cerevisiae the RNA polymerase III transcription factor TFIIIC binds to sites where binding of other components of the RNA polymerase III machinery is not detected and where the transcription factor does not activate transcription.

### Identification of Putative SpoIIID-Binding Sites by Bioinformatics

**Figure 4:**
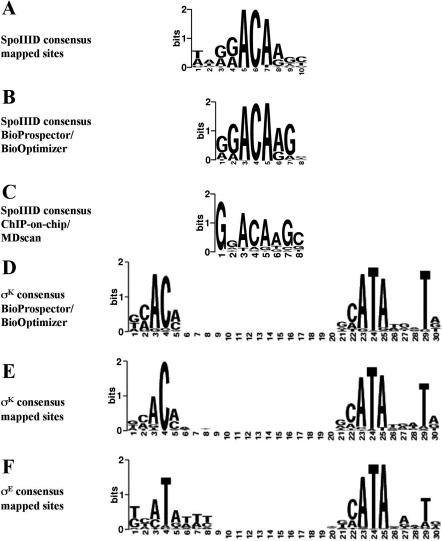
Consensus Sequences for SpoIIID, σ^K^, and σ^E^ Consensus sequences are displayed as sequence logos (Schneider and Stephens 1990). The height of the letters in bits represents the information content at each position (the maximum value is two bits). (A) Consensus binding sequence for SpoIIID as derived from 17 SpoIIID-binding sites mapped by DNAase I footprinting (Halberg and Kroos 1994; Zhang et al 1997; results presented herein). (B) Consensus binding sequence for SpoIIID obtained by compilation of 68 putative SpoIIID-binding sites identified as common motifs by BioProspector and BioOptimizer analysis in sequences upstream of genes identified by transcriptional profiling or within regions identified by ChIP-on-chip analysis. (C) Consensus binding sequence for SpoIIID obtained by MDscan analysis of the sequences of 26 SpoIIID-binding regions identified by ChIP-on-chip analysis. (D) Consensus promoter sequence for σ^K^-containing RNA polymerase obtained from the compilation of 58 sequences identified as common motifs in regions upstream of σ^K^-regulated genes by a BioProspector/BioOptimizer computational approach (Jensen and Liu 2004). Positions 1–5 on the horizontal axis correspond to the −35 element and positions 21–30 to the −10 element. The optimal spacing between the two regions is 15 bp (± 1 bp). (E) Consensus promoter sequence for σ^K^-containing RNA polymerase obtained from the compilation of 23 previously mapped (http://dbtbs.hgc.jp/; Helmann and Moran 2002) and 18 newly identified σ^K^-controlled promoters identified by transcription start site mapping. (F) Consensus promoter sequence for σ^E^-containing RNA polymerase obtained from the compilation of 62 σ^E^-controlled promoters identified by transcription start site mapping (Eichenberger et al. 2003). Positions 1–8 on the horizontal axis correspond to the −35 element, and positions 21–30 to the −10 element. The optimal spacing between the two regions is 12 bp (± 1 bp).

As a final, computational approach to identifying direct targets of SpoIIID, we used the Gibbs sampling algorithm BioProspector to identify conserved motifs in sequences upstream of genes under the control of SpoIIID (Liu et al. 2001). Initially, we limited our search to 40 regions where SpoIIID binding had been confirmed by biochemical analysis. BioProspector was used to find the best 35 motifs across several different widths (6–12 bp) under the restriction that every sequence had to contain at least one site. Each of these motifs was separately used as a starting point for BioOptimizer (Jensen and Liu 2004) and applied to an expanded dataset that included the 89 upstream sequences for all SpoIIID-controlled genes (not just those analyzed by EMSA or footprinting). BioOptimizer optimized both the set of predicted sites and the motif width, as detailed in the Materials and Methods section. BioOptimizer was required to identify at least one binding site in the sequences that had been confirmed by EMSA but was unrestricted for the sequences for which a binding site had not been confirmed biochemically. The optimized motif was 8 bp in length and identified at least one putative SpoIIID-binding site in 60 of the 89 upstream sequences that were analyzed (see Table S2). Figure 4 shows that the logo for the optimized motif (B) was similar to a consensus sequence (A) that was derived independently using 12 previously reported binding sites (for the genes *bofA, cotD, spoVD, spoIVCA,* and *spoIVCB;* Halberg and Kroos 1994; Zhang et al. 1997) and five sites herein identified by DNAase I footprinting.

In an independent computational approach, we sought to identify a conserved motif in the 26 regions that had been identified by ChIP-on-chip analysis, which likely represent the strongest binding sites for SpoIIID. We used Motif Discovery scan(MDscan) (Liu et al. 2002) for this analysis, which is designed to identify conserved motifs in sequences that have been ranked according to their enrichment factor in ChIP-on-chip experiments. The resulting sequence logo is displayed in Figure 4C. Whereas it is largely similar to that obtained from the BioProspector/BioOptimizer analysis (Figure 4B), there is one notable difference: The first position of the binding motif corresponds almost exclusively to a guanine in the sites identified by ChIP-on-chip analysis. The presence of a guanine at this position could be characteristic of high-affinity sites for SpoIIID binding.

In conclusion, SpoIIID negatively or positively influences the transcription of over half of the members of the σ^E^ regulon, and a combination of complementary approaches leads us to believe that it does so for many of the genes so identified by direct interaction with their promoter regions. In the case of genes under the negative control of SpoIIID, the mechanism of this repression probably involves steric interference as the inferred binding sites for SpoIIID were generally found to overlap with the expected binding sites for RNA polymerase. No such overlap was generally observed in the case of genes under the positive control of SpoIIID.

### GerR *(ylbO),* a Second Negative Regulator of the σ^E^ Regulon

The *spoIIID* gene is not the only member of the σ^E^ regulon that appears to encode a DNA-binding protein. The inferred product of *ylbO* exhibits significant similarity to members of the basic leucine zipper family of transcription factors and is, in particular, 52% similar to RsfA (Wu and Errington 2000), a regulator of σ^F^-controlled genes in the forespore line of gene expression. To study a possible role for *ylbO* we investigated the effect of a null mutation of the gene on sporulation and on σ^E^-directed gene expression. As noted previously, the mutation has no effect on the production of heat-resistant spores, but we have now discovered that the mutation causes a conspicuous defect in the capacity of the spores to germinate, as judged by their impaired ability to reduce 2,3,5-triphenyltetrazolium chloride (see Materials and Methods). We therefore rename *ylbO* as *gerR* (in keeping with the nomenclature for germination genes in B. subtilis [Setlow 2003]). We also carried out transcriptional profiling using RNA collected at hour 3.5 of sporulation from cells of a strain (PE454) that was wild type for GerR and from cells of a newly constructed strain (SW282) that was mutant for GerR. Both strains were also mutant for the next transcription factor in the hierarchical cascade, σ^K^. No genes were identified whose transcription was dependent on GerR, but 139 genes were found that were downregulated in a GerR-dependent manner by a factor of two or greater (see Table S1). Among the downregulated genes were 14 members of the σ^E^ regulon. Nine of these members (colored blue in Figure 2A) were known not to be under SpoIIID control *(cypA, kapD, spoIIM, spoIIP, ybaS , yfnE , yfnD, yhjL,* and *yqhV),* whereas the remaining five *(phoB, spoIIIAA, spoIIIAB, spoIVCA,* and *ydhF)* were also under the control of SpoIIID.

**Figure 5:**
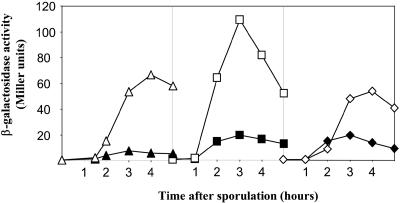
Repression of σ^E^ -Controlled Genes by GerR Culture samples from strains PE551 (solid triangles, *amyE*::P*_spoIIM_–lacZ*), SW312 (open triangles, *amyE*::P*_spoIIM_–lacZ,* Δ*gerR*), PE511 (solid squares, *amyE*::*spoIIP–lacZ*), PE568 (open squares, *amyE*::*spoIIP–lacZ,*Δ*gerR*), PE553 (solid diamonds, *amyE*::P*_yqhV_–lacZ*), and PE558 (open diamonds, *amyE*::P*_yqhV_–lacZ,* Δ*gerR*) were collected at indicated intervals after the start of sporulation in Sterlini–Mandelstam medium and analyzed for β-galactosidase activity.

We selected three of the putative targets of GerR for further analysis. The promoter sequences of *spoIIM* and *yqhV* were fused to the coding sequence of β-galactosidase and introduced into the chromosome at the *amyE* locus and a previously constructed fusion of *lacZ* to *spoIIP (amyE*::*spoIIP*-*lacZ)* was obtained from P. Stragier (Institut de Biologie Physico-Chimique, Paris). The results, shown in Figure 5, confirmed that GerR had a pronounced negative effect on the level of expression of all three fusions.

An example of σ^E^-controlled genes that are under the dual negative control of GerR and SpoIIID is the eight-cistron *spoIIIA* operon (Illing and Errington 1991). As we have demonstrated, GerR is responsible for repressing *yqhV,* which is located just upstream of the *spoIIIA* operon. Given the absence of an apparent transcriptional terminator at the end of the gene, σ^E^-directed transcription from *yqhV* is likely to read into *spoIIIA,* which is also transcribed from its own σ^E^-controlled promoter located in the intergenic region between *yqhV* and the operon. Thus, by repressing *yqhV,* GerR would inhibit read-through transcription into *spoIIIA*. Indeed, our transcriptional-profiling analysis revealed a small negative effect of GerR on *spoIIIA* transcription. Meanwhile, SpoIIID acts at the promoter for the *spoIIIA* operon to inhibit it from being used by σ^E^-RNA polymerase. Thus, maximum repression of *spoIIIA* is evidently achieved by the combined action of GerR and SpoIIID, each acting to block different promoters.

Finally, we note that GerR inhibited the expression of a large number of genes that do not belong to the σ^E^ regulon. Interestingly, many of these genes are organized in large clusters, such as *azlB–azlC–azlD–bnrQ–yrdK–gltR, albA–albB–albC–albD, yefA–yefB–yefC–yeeA–yeeB–yeeC, yjcM–yjcN–yjcO,* and *yydB–yydC–yydD–yydG–yydH–yyd –yydJ*. The genes found in these clusters rarely belong to a single transcription unit and are sometimes transcribed in opposite directions (either convergently or divergently).

In summary, transcription of genes in the σ^E^ regulon is in part self-limiting. The σ^E^ factor induces the synthesis of two proteins, GerR and SpoIIID, that act to switch off other genes in the regulon, thereby preventing their continued transcription during the next stage of the mother-cell line of gene expression.

### The σ^K^ Regulon

Next, we used two complementary transcriptional-profiling approaches to identify genes under the control of σ^K^, an RNA polymerase sigma factor that follows SpoIIID in the hierarchical regulatory cascade. In one approach, we sought to identify genes that were upregulated during sporulation in a σ^K^-dependent (but not a GerE-dependent) manner. In the other approach, we sought to identify genes whose transcription was artificially activated in cells engineered to produce σ^K^ during growth. For this approach we used a strain in which the coding sequence for the mature form of the transcription factor (σ^K^ is normally derived by proteolytic processing from an inactive proprotein [Kroos et al. 1989]) was under the control of an inducible promoter (see Materials and Methods). Ninety-five genes were identified that were induced both during growth and sporulation in a σ^K^-dependent manner. Eight additional genes *(cotA, cotE, cotM*, *gerE, gerPA, yfhP, yjcZ*, and *ykuD)* that had previously been assigned to the regulon on the basis of gene-specific analysis were added to the tally, bringing the total to 103 (and representing 63 transcription units). These eight genes were cases in which we did not obtain a statistically significant score in one or the other of the two transcriptional-profiling approaches or for which a signal was not obtained for technical reasons (e.g., the strain used was mutant for *gerE* and *yjcZ* had not been annotated when the arrays were built). The list of 103 did not include σ^K^-controlled genes whose transcription additionally and strongly required the DNA-binding protein GerE. Some (28) of these 103 genes were also transcribed under the control of σ^E^ (see Table 2), leaving a total of 75 genes that were newly activated during sporulation under the control of σ^K^. As we shall see, when genes that were strongly dependent on GerE are included (41 genes, five of which were also expressed under the control of σ^E^), the size of the regulon increases to 144 genes (103 + 41) organized in 94 transcription units (Table 2). A map of the σ^K^ regulon is displayed in Figure 2B and a detailed list of the genes in the regulon is presented in Table S4.

### Identification of Promoters Controlled by σ^K^ Using Bioinformatics and Transcriptional Start Site Mapping

As a further approach to assessing our assignments to the σ^K^ regulon, we used BioProspector and BioOptimizer to obtain a consensus sequence for promoters under the control of the sporulation transcription factor. The computational approach was complicated by the fact that the program had to find a two-block motif, with the first block corresponding to the −35 element and the second block to the −10 element separated by a gap of fixed length (+/− one nucleotide). The dataset consisted of 76 upstream sequences (the upstream sequences of transcription units that were strongly dependent on GerE were not included). The optimized motif with the best score identified 58 promoters and was composed of a five-nucleotide-long −35 element and a ten-nucleotide long −10 element, separated by a gap of 14–16 nucleotides (Figure 4D; Jensen and Liu 2004) To assess the validity of the predicted consensus sequence for σ^K^ promoters, we mapped the transcription start sites of 18 of the newly identified targets of σ^K^ by 5′ rapid amplification of complementary DNA ends–PCR (RACE–PCR). The results of the mapping experiments are displayed in Figure S2. The newly identified σ^K^ promoters were combined with the promoter sequences of 23 previously mapped σ^K^ promoters to obtain an updated consensus sequence corresponding to a total of 41 promoters (Figure 4E). The logo for σ^K^ promoters whose start sites had been mapped was very similar to the logo obtained by the BioProspector/BioOptimizer procedure (see Figure 4D). Moreover, out of the 41 confirmed σ^K^ promoters, the correct promoter was identified in 24 cases, with no prediction being made in 15 cases and an incorrect prediction in just two cases. All of the predicted sites are listed in Table S4.

The σ^E^ and σ^K^ factors are highly similar to each other, and the promoters they recognize are also very similar. The availability of updated logos for both categories of promoters based on the nearly complete regulons for both regulatory proteins provided an opportunity to revisit the issue of how the two regulatory proteins discriminate between their two classes of cognate promoters. A comparison of the motif recognized by σ^K^ to that recognized by σ^E^ (Figure 4F) reveals that both classes of promoters share identical −10 sequences and that the −35 elements differ by a single base pair: a cytosine in the fourth position of σ^K^-controlled promoters versus a thymine at the corresponding position in σ^E^-controlled promoters. These results reinforce the findings of Tatti et al. (1995) who identified glutamine 217 of σ^E^ as the contact residue for the base pair at position 4. The two proteins are identical to each other in the region inferred to interact with the −35 element except for the presence of arginine instead of glutamine at the corresponding position in σ^K^. Moreover, replacing glutamine 217 with arginine was found to confer on σ^E^ the capacity to recognize σ^K^-controlled promoters (Tatti et al. 1995). The high similarity between the two classes of promoters also helps to explain why some σ^K^-controlled promoters are also recognized by σ^E^, but our bioinformatics analysis does not allow us to explain why some promoters are recognized exclusively by one or the other sigma factor and others are not.

### GerE Is Both a Repressor and an Activator of Genes Whose Transcription Is Dependent upon σ^K^

The last regulator in the mother-cell line of gene expression is the DNA-binding protein GerE (Cutting et al. 1989). Genes under GerE control were identified by transcriptional-profiling experiments carried out at two times (5.5 h and 6.5 h) late in sporulation. Strikingly, as many as 209 genes were downregulated in the presence of GerE at one or both time points, with many more genes being downregulated at the later time point (201 versus 61; see Table S1). Some of these downregulated genes (55) were members of the σ^K^ regulon, with 29 being downregulated at the earlier time point and an additional 26 at the later time point. Thus, GerE is responsible for inhibiting the expression of 53% of the genes in the σ^K^ regulon, but its repressive effects are not limited to genes under σ^K^. We note that the gene coding for σ^K^ is itself repressed by GerE, which would be expected to curtail further synthesis of the mother-cell sigma factor late in sporulation. Thus, GerE has a wide impact in inhibiting gene transcription late in the process of spore maturation, including many genes in the preceding regulon of σ^K^-activated genes.

At the same time, GerE is also an activator that stimulated or switched on the transcription of as many as 65 genes by hour 5.5 and 71 genes by hour 6.5. Of these, 41 were strongly dependent upon GerE for their expression and hence were not identified as members of the σ^K^ regulon. Leaving aside genes that were members of both the σ^E^ and σ^K^ regulons (five), we see that GerE is responsible for turning on an additional 36 genes (27 transcription units) in the final phase of the mother-cell line of gene expression (Table 2).

### Evidence that SpoIIID-Mediated Repression Is Required for Sporulation

As we have seen, a striking feature of the mother-cell line of gene expression is that many of the genes activated by one transcription factor are turned off by the next-appearing regulatory protein in the cascade. Thus, most of the genes that are turned on by σ^E^ are subsequently repressed by GerR or SpoIIID. Likewise, many of the genes activated by σ^K^ are, in turn, downregulated by GerE. In the case of GerR, a mutant lacking the regulatory protein produced spores that were defective in germination. Hence, proper morphogenesis depends on the capacity of GerR, which appears to act exclusively as a repressor, to turn off genes under its control.

The case of SpoIIID is more complex because in addition to its role as a repressor this DNA-binding protein is also an activator of two genes, *spoIVCA* and *spoIVCB,* that are essential for sporulation because of their role in the synthesis of σ^K^ (Halberg and Kroos 1994). To investigate the role of SpoIIID-mediated repression in spore formation, we created a construct in which a copy of the intact pro-σ^K^ coding sequence, *sigK,* was introduced into the *amyE* locus, thereby bypassing the requirement for the *spoIVCA*-encoded recombinase, which is normally needed for creating *sigK* by a chromosomal rearrangement (Stragier et al. 1989), and for *spoIVCB,* the 5′ portion of the coding sequence that participates in the rearrangement. In our construct, the insertion of *sigK* at *amyE* was under the direction of a σ^E^-controlled promoter that is not dependent upon SpoIIID for its activation (the promoter for *spoIVF;* Cutting et al. 1991b). The *amyE*::P*_spoIVF_–sigK* construct was introduced into *spoIVCB* mutant cells to create strain BDR1663. Even though pro-σ^K^ was expected to be synthesized somewhat prematurely in BDR1663, the appearance of mature σ^K^ remained subject to the pathway governing the proteolytic processing of pro-σ^K^ and hence would have occurred at the normal time (Cutting et al. 1991a). Indeed, cells harboring the *amyE*::P*_spoIVF_–sigK* construct sporulated as efficiently as the wild type and did so in a manner that did not depend on the presence of *spoIVCB* (Table 3). We conclude that bypassing the requirement for SpoIIID in σ^K^ synthesis does not measurably affect sporulation efficiency.

**Table 3:**
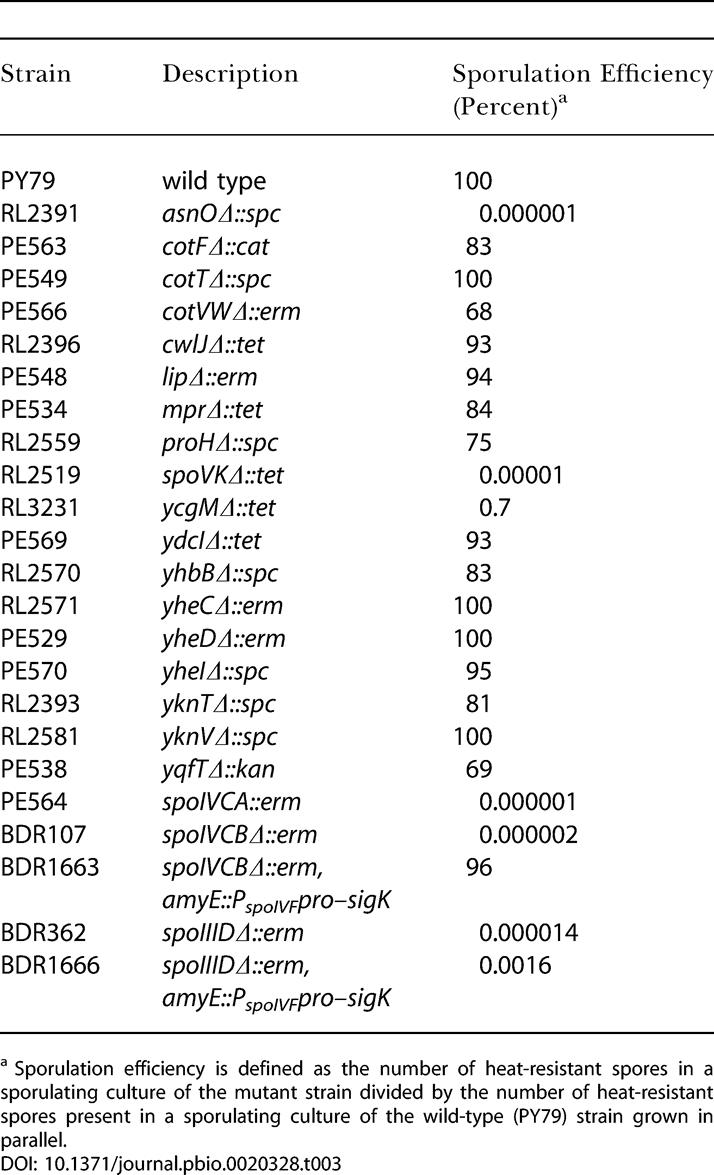
Systematic Inactivation of SpoIIID-Activated Genes

^a^ Sporulation efficiency is defined as the number of heat-resistant spores in a sporulating culture of the mutant strain divided by the number of heat-resistant spores present in a sporulating culture of the wild-type (PY79) strain grown in parallel

However, when the *amyE*::P*_spoIVF_–sigK* construct was introduced into cells harboring a *spoIIID* mutation (generating strain BDR1666), sporulation efficiency was still reduced by about a 100,000-fold compared to the wild type (Table 3). This result reinforces the findings of Lu and Kroos (1994), who showed that sporulation was impaired in *spoIIID* mutant cells even in the presence of a construct that allowed pro-σ^K^ to be produced in a SpoIIID-independent manner. A possible explanation for these results is that, in addition to its role in σ^K^ synthesis, SpoIIID is required for the synthesis of some other unidentified protein or proteins that are needed for sporulation. To investigate this possibility, we systematically inactivated all of the newly identified SpoIIID-activated transcription units (Table 3). With three exceptions, those of *spoVK, asnO,* and *ycgM,* the resulting mutants sporulated at levels comparable to that of the wild type. In the case of *spoVK, asnO,* and *ycgM,* evidence suggests that each is transcribed in both a SpoIIID-dependent and a SpoIIID-independent mode. Thus, *spoVK* is transcribed from both a σ^E^-controlled (P1) and a σ^K^-controlled (P2) promoter, and it is known that P1 is dispensable for sporulation (Foulger and Errington 1991). Experiments based on the use of cells engineered to produce σ^K^ during growth indicate that *asnO* is capable of being transcribed under the direction of σ^K^. Finally, it has been shown that *ycgM* is induced during the early stages of sporulation under the control of Spo0A (Molle et al. 2003a), and so at least some YcgM protein should be present in a *spoIIID* mutant. Besides, complete inactivation of *ycgM* resulted in a sporulation defect that is less severe than that observed for strain BDR1666.

These results do not rule out the possibility that SpoIIID activates the transcription of one or more genes in addition to *spoIVCA* and *spoIVCB* that are needed for sporulation. Nevertheless, the simplest interpretation of our findings is that the strong sporulation defect of strain BDR1666 is due to a failure in gene turn off rather than gene activation.

## Discussion

### The Mother-Cell Line of Gene Transcription Is a Hierarchical Regulatory Cascade That Is Subject to Successive Negative Regulatory Loops

Our results reveal the almost complete program of gene transcription for a single differentiating cell type, the mother-cell compartment of the B. subtilis sporangium. The mother cell is a terminally differentiating cell that ultimately undergoes lysis (programmed cell death) when its contribution to the maturation of the spore is complete. Its program of transcription is played out over the course of about 5 h and, as we have shown, involves the activation in a cell-type-specific manner of 383 genes, which are grouped together in 242 transcription units. This corresponds to 9% of the 4,106 annotated protein-coding genes in the B. subtilis genome. The transcription of these 383 genes is orchestrated by five developmental regulatory proteins: two RNA polymerase sigma factors, σ^E^ and σ^K^, and three DNA-binding proteins, SpoIIID, GerE, and a previously uncharacterized regulatory protein, GerR. The five regulatory proteins are organized in a hierarchical regulatory cascade of the form: σ^E^→SpoIIID/GerR→σ^K^ →GerE. The earliest-acting regulatory protein in the cascade, σ^E^, turns on the transcription of 262 genes (163 transcription units), including the genes for GerR and SpoIIID. GerR and SpoIIID, in turn, acting as repressors, downregulate further transcription of almost half of the genes in the σ^E^ regulon. In addition, however, SpoIIID, acting in conjunction with σ^E^-containing RNA polymerase, turns on the transcription of ten genes (eight transcription units), including genes involved in the appearance of σ^K^. Next, σ^K^ activates 75 additional genes (44 transcription units). Among the members of the σ^K^ regulon is the gene for the final regulatory protein in the cascade GerE. Strikingly, GerE represses the transcription of over half of the genes that have been activated by σ^K^ while switching on 36 additional genes (27 transcription units), the final temporal class in the mother-cell line of gene transcription. Thus, the program of gene expression is driven forward by its hierarchical organization as well as by the successive, repressive effects of the DNA-binding proteins, which inhibit continued transcription of many genes that had been activated earlier in the cascade. Indeed, evidence presented herein is consistent with the idea that repression by GerR and SpoIIID contributes to proper sporulation, modestly in the case of GerR, and perhaps more significantly in the case of SpoIIID.

### The Mother-Cell Line of Gene Transcription Is Governed by a Linked Series of Coherent and Incoherent FFLs

Transcription networks are based on recurring circuit modules, one of the most common of which is the FFL (Milo et al. 2002; Shen-Orr et al. 2002; Mangan and Alon 2003). FFLs are simple circuits involving two regulatory proteins in which one (the primary regulatory protein) governs the synthesis of the other and both then control the expression of a set of target genes. Certain types of FFLs known as type 1 are particularly prevalent because of their favorable biological properties (Shen-Orr et al. 2002). In type-1 FFLs, the primary regulatory protein acts positively on the synthesis of the second. The mother-cell line of gene transcription is based on two kinds of type-1 FFLs known as a “coherent” and “incoherent.” In coherent type-1 FFLs, both regulatory proteins act positively on target genes, whereas in incoherent type-1 FFLs, the primary regulatory protein acts positively and the second acts negatively.

Using this nomenclature, we see that the hierarchical regulatory cascade that governs the mother-cell line of gene transcription is a circuit composed of two coherent type-1 FFLs linked in series (Figure 1B). Thus, σ^E^ turns on the synthesis of SpoIIID, and both transcription factors then act jointly to turn on target genes, including genes involved in the appearance of σ^K^. The FFL is acting by the logic of an AND gate in that both σ^E^ and SpoIIID are required for the expression of target genes. This first FFL is linked in series to a second coherent type-1 FFL in which σ^K^ turns on the synthesis of GerE, and the two transcription factors then collaborate to activate the transcription of target genes (the terminal temporal class of gene transcription in the mother cell). Once again this is an AND gate in that both σ^K^ and GerE are required for the activation of target genes. Simulation studies show that coherent type-1 FFLs have the property of being persistence detectors in which the activation of target genes depends on the persistence of the primary regulatory protein (σ^E^ and σ^K^) and “rejects” situations in which the primary regulatory protein is present only transiently in its active form (Mangan and Alon 2003).

The mother-cell line of gene transcription is also governed by three incoherent type-1 FFLs, involving SpoIIID, GerR, and GerE, each acting in this context as repressors. Thus, σ^E^ turns on the synthesis of SpoIIID, which in turn represses a subset of the genes that have been turned on by the primary regulatory protein. The σ^E^ factor similarly turns on the synthesis of GerR, which then represses a largely nonoverlapping subset of the genes that have been activated by σ^E^. Finally, the σ^K^ factor turns on the synthesis of GerE, which then acts to downregulate the transcription of many of the genes that have been switched on by σ^K^. Simulations have shown that incoherent type-1 FFLs have the property of producing a pulse of gene transcription (Mangan and Alon 2003). Incoherent type-1 FFLs also operate by the logic of an AND gate in that pulses of gene transcription require the action of both the activator and the delayed appearance of the repressor.

Viewing the mother-cell line of gene transcription in terms of an interconnected series of FFLs reveals an underlying logic to the mother-cell program of gene expression. The use of coherent type-1 FFLs to drive the activation of successive sets of genes and the ordered appearance of regulatory proteins may help to minimize noise and to ensure that each temporal class of gene activation is tightly tied to the persistence of the previously acting regulatory proteins in the sequence (Mangan et al. 2003). Meanwhile, the use of incoherent type-1 FFLs to switch off the transcription of genes in previously activated gene sets helps to generate pulses of gene transcription in which certain genes, whose products may only be required transiently during differentiation, are transcribed over a limited period of time. Indeed, as we now consider, genes with related functions are often transcribed coordinately in a pulse, the timing of which corresponds to the function of their products.

### Coordinated Expression of Functionally Related Genes

The mother-cell program of gene expression is characterized, as we have seen, by pulses of gene expression in which different sets of genes are successively switched on and then switched off. In some cases, these pulses correspond to the expression of genes with related functions (Table 4). This can be most clearly seen with the gene set that is activated by σ^E^ and repressed by SpoIIID or GerR, which includes genes involved in engulfment, cortex formation, and the appearance of σ^G^ and σ^K^. Thus, three genes that are responsible for driving engulfment, *spoIID* (Lopez-Diaz et al. 1986), *spoIIM* (Smith and Youngman 1993; Smith et al. 1993), and *spoIIP* (Frandsen and Stragier 1995), are coordinately activated by σ^E^ and then repressed by SpoIIID (in the case of *spoIID*) or by GerR (in the case of the other two). Likewise, all of the σ^E^-controlled genes that are known to be required for spore cortex formation (*cwlD, dacB–spmAB, spoIVA, spoVB, spoVD, spoVE, yabPQ, ykvUV, ylbJ,* and *yqfCD;* Piggot and Losick 2002; Eichenberger et al. 2003) are repressed by SpoIIID. Yet another example is the eight-gene *spoIIIA* operon, which is involved in the activation of σ^G^ in the forespore (Stragier and Losick 1996). The operon is transcribed from two σ^E^-controlled promoters, one located immediately upstream of the operon and one preceding the next upstream gene, *yqhV*. As we have shown, both promoters are turned off shortly after their activation; one by SpoIIID and the other by GerR.

**Table 4:**
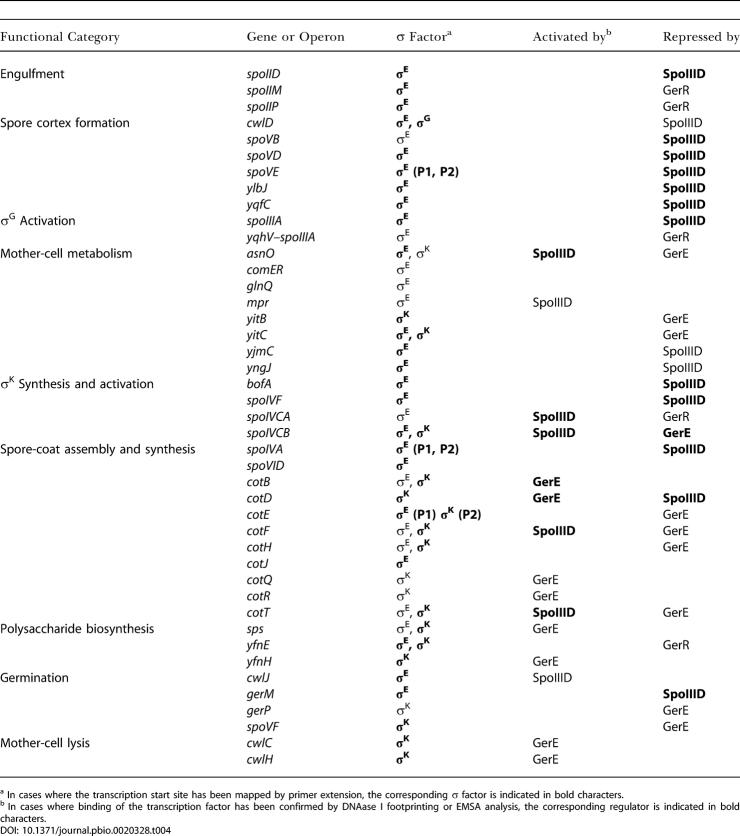
Functional Categories

^a^ In cases where the transcription start site has been mapped by primer extension, the corresponding σ factor is indicated in bold characters

^b^ In cases where binding of the transcription factor has been confirmed by DNAase I footprinting or EMSA analysis, the corresponding regulator is indicated in bold characters

Particularly illuminating is the case of the five σ^E^-controlled genes involved in the appearance of σ^K^: *bofA, spoIVCA, spoIVCB, spoIVFA,* and *spoIVFB*. Two of these genes *(spoIVCA* and *spoIVCB)* are involved in the synthesis of the proprotein precursor, pro-σ^K^, whereas the remaining three *(bofA*, *spoIVFA,* and *spoIVFB)* are involved in the conversion of the proprotein to mature σ^K^ (Cutting et al. 1991b; Ricca et al. 1992). Interestingly, *bofA*, *spoIVFA,* and *spoIVFB* are repressed by SpoIIID, whereas *spoIVCA* and *spoIVCB* are switched on by SpoIIID, in this context acting as an activator. Hence, and ironically, genes involved in the processing of pro-σ^K^ are expressed in a pulse that precedes the time of activation of the genes involved in the synthesis of the substrate for processing.

How can we explain these seemingly anomalous observations? BofA, SpoIVFA, and SpoIVFB are integral membrane proteins that form a complex in the mother-cell membrane that surrounds the forespore (Resnekov et al. 1996; Rudner and Losick 2002). Evidence indicates that they initially localize to the cytoplasmic membrane that surrounds the mother cell and then reach their final destination by diffusion to, and capture at, the outer forespore membrane (Rudner et al. 2002). Such a diffusion-and-capture mechanism requires that the synthesis of BofA, SpoIVFA, and SpoIVFB takes place prior to the completion of engulfment since the outer membrane surrounding the forespore has become topologically isolated from the cytoplasmic membrane once engulfment is complete. Conversely, no such restriction applies to pro-σ^K^ (a peripheral membrane protein) whose synthesis is delayed (by virtue of being under the positive control of SpoIIID) relative to that of the integral membrane proteins. Strikingly, and in extension of these observations, a high proportion of σ^E^-controlled genes that encode proteins with predicted transmembrane segments are negatively regulated by SpoIIID and GerR. We speculate that many of these genes encode proteins that localize to the outer forespore membrane and do so by a diffusion-and-capture mechanism. Hence their synthesis is restricted to the time prior to the completion of engulfment. By contrast, σ^E^-controlled genes that are unaffected by SpoIIID and GerR, or are activated by SpoIIID, rarely encode proteins with predicted transmembrane segments (see Table S2).

As a final example of the coordinate expression of genes with related function we consider the case of *cwlC* and *cwlH,* which are switched on in the terminal phase of differentiation under the positive control of GerE (Kuroda et al. 1993; Smith and Foster 1995; Nugroho et al. 1999). The *cwlC* and *cwlH* genes encode cell-wall hydrolases that are responsible for the lysis of the mother cell when morphogenesis is complete so that the mature spore can be liberated from the sporangium. It is of crucial importance that mother-cell lysis not take place prematurely, and thus it makes sense that genes involved in this process are among the last genes to be turned on in the mother-cell line of gene expression.

### Some Functionally Related Gene Classes Exhibit Heterogeneous Patterns of Gene Expression

Many of the genes in the mother-cell line of gene expression are known or inferred to be involved in metabolism, assembly of the spore coat, or the synthesis of coat-associated polysaccharides (see Table 4). Interestingly, not all of the genes in these categories are coordinately expressed. Rather, genes in all three categories exhibit heterogeneous patterns of expression. Thus, among genes inferred to be involved in metabolism, some, such as members of the *yngJIHGFE* operon, which are expected to govern lipid catabolism, and members of the *yjmCD–uxuA–yjmF–exuTR* operon, which are expected to direct hexuronate synthesis (Mekjian et al. 1999), are expressed early in development, whereas other genes, such as the members of the *yitCD* and *yitBA*–*yisZ* operons, which are inferred to be involved in phosphosulfolactate synthesis (Graham et al. 2002), are expressed late in development. Sulfolactate is indeed known to be a major component of the dry weight (5%) of mature spores of B. subtilis but is not found in spores of B. megaterium and B. cereus (Bonsen et al. 1969). Consistent with these observations, the genome of B. cereus lacks an ortholog of the *yitCD* operon. Interestingly, the gene for *asnO,* which encodes an asparagine synthetase (Yoshida et al. 1999), is under the positive control of three of the five mother-cell-specific transcription factors (σ^E^, σ^K^, and SpoIIID) and the negative control of GerE, and hence its expression is maintained until very late in development.

Of special interest are genes involved in the assembly of the coat, the most conspicuous morphological feature of the mature spore. The coat is a complex, two-layered structure that creates a protective shield around the spore and is composed of at least 30 proteins (Driks 2002; Kuwana et al. 2002; Takamatsu and Watabe 2002; Lai et al. 2003). The earliest-acting protein in the formation of the coat is SpoIVA, which creates a substratum around the outer forespore membrane upon which assembly of the coat takes place (Roels et al. 1992; Stevens et al. 1992; Driks et al. 1994; Price and Losick 1999). In keeping with its early role in the assembly process, the gene for SpoIVA is switched on early in the mother-cell line of gene expression under the control of σ^E^ and is then turned off by the action of SpoIIID. The σ^E^ factor also turns on the genes for at least five other coat proteins that play important roles in coat assembly (*cotE, cotH, safA, spoVM,* and *spoVID;* Piggot and Losick 2002)*,* but expression of these genes persists longer than that for *spoIVA* as none of these is repressed by SpoIIID. In fact, *cotE* and *cotH* continue to be expressed at even higher levels later in development under the control of σ^K^, eventually being downregulated by GerE. In the case of *cotE,* Li and Piggot (2001) have shown that transcription from its σ^E^-dependent promoter P1 ceases before the activation of σ^K^. Interestingly, certain temporal classes of mother-cell-specific genes are particularly enriched in coat protein genes. For instance, almost half of the σ^E^-controlled genes that are strongly or partially dependent on SpoIIID for expression (i.e., ten out of 25; C. F., P. E., and R. L., unpublished data) code for coat proteins. Similarly, our preliminary cytological data (C. F., P. E., and R. L., unpublished data) indicate that many of the newly identified σ^K^-controlled genes encode coat-associated proteins.

In addition to being composed of many different proteins, the coat is composed of polysaccharides. Playing an important role in the synthesis of these polysaccharides is the 11-gene *sps* operon, the longest of the 236 mother-cell-specific transcription units identified in this study. The *sps* operon is transcribed from a σ^K^-controlled promoter, which we have mapped to a site just upstream of the first gene in the operon, *spsA*. Transcription from this promoter is enhanced by the appearance of GerE but is not dependent upon it. Thus, expression of genes involved in the biosynthesis of spore-coat polysaccharides persists until the very late stages of sporulation, in keeping with the idea that these polysaccharides are a component of the outer surface of the spore. Nevertheless, some genes in the *sps* operon are switched on early in sporulation under the control of σ^E^, most likely from a second promoter located upstream of the seventh gene in the operon, *spsG*. Hence *spsG* and the genes downstream of it exhibit a protracted pattern of expression that persists throughout the entire process of differentiation.

The *sps* operon may not be the only set of genes involved in the synthesis of coat-associated polysaccharides. We have identified several paralogs of members of the operon that contribute to the mother-cell line of gene expression. These include genes in the *yfnED* operon, which is switched on by σ^E^, downregulated by GerR, and turned on again by σ^K^. Another example is the *yfnHGF* operon, which is under the positive control of σ^K^ and GerE. Yet another example is a paralog of *spsJ, yodU–ypqP,* which is activated under the dual control of σ^K^ and GerE. Interestingly, in the strain used in this study (PY79), *yodU* and *ypqP* actually correspond respectively to the 5′ end and the 3′ end of a single gene. However, in strain 168, the gene formed by *yodU* and *ypqP* is interrupted by the prophage of the large temperate phage SPβ, thereby greatly separating *ypqP* from the sporulation promoter that would otherwise direct its transcription. It would be interesting to investigate whether the interruption of the *yodU*–*ypqP* gene by SPβ influences the polysaccharide composition of the spore coat.

### The Mother-Cell Line of Gene Transcription in Other Endospore-Forming Bacteria

Endospore formation has been documented in many species of the low G+C group of gram-positive bacteria (Stragier 2002). Two distantly related genera in that group, *Bacillus* and *Clostridium,* are able to sporulate, whereas several genera that are phylogenetically closer to *Bacillus,* such as *Listeria* and *Staphylococcus,* do not sporulate. Remarkably, in genome regions of otherwise high conservation (synteny) to corresponding regions in *B. subtilis,* sporulation genes are missing from *Listeria* (Eichenberger et al. 2003) and *Staphylococcus.* It is likely that the common ancestor of all of these genera was an endospore-forming bacterium and that sporulation genes were deleted over time from genera that had adapted alternative modes of survival in their ecological niche or host in a manner that did not involve the need for a robust resting state.

To investigate further the evolutionary relatedness of the mother-cell differentiation program among endospore-forming species, we searched for the presence of orthologs of B. subtilis genes in the mother-cell line of gene expression in the genome sequences of the following species: B. anthracis (Ames strain) (Read et al. 2003), B. cereus (ATCC14579) (Ivanova et al. 2003), B. halodurans (Takami et al. 2000), and Oceanobacillus iheyensis (HTE831) (Takami et al. 2002); Listeria monocytogenes and L. innocua (Glaser et al. 2001); and Clostridium acetobutylicum (ATCC 824) (Nolling et al. 2001) and C. perfringens (strain 13) (Shimizu et al. 2002) (Tables S2 and S4).

First, we searched for orthologs of the mother-cell-specific transcription factors. Interestingly, whereas genes for σ^E^, σ^K^, and SpoIIID were present in the *Bacillus* and *Clostridium* species, GerE (Stragier 2002) and GerR were absent from *Clostridium,* suggesting that significant differences exist in the mother-cell programs between the two genera, especially during the terminal (GerE-controlled) phase of gene expression. Nonetheless, in cases when a transcription factor was conserved between *Bacillus* and *Clostridium,* the protein domains involved in nucleotide-sequence recognition were also highly conserved, indicating that the consensus binding sequences that we described here are likely to be conserved among many, if not all, endospore-forming bacteria. For instance, the glutamine residue that recognizes the specificity determinant in the −35 element of σ^E^-controlled promoters is absolutely conserved in all of the available σ^E^ protein sequences, and the corresponding arginine is conserved in all of the available σ^K^ protein sequences.

In addition to differences in the presence of certain mother-cell regulatory proteins (e.g., GerR and GerE) among endospore-forming species, the gene composition of the individual regulons also varies in a species-specific manner. In general, genes in the σ^E^ regulon appear to be more highly conserved than genes in the σ^K^ regulon. For instance, approximately 75% of the B. subtilis σ^E^-controlled transcription units have orthologs in B. anthracis and *B. cereus,* whereas only 50% of the σ^K^-controlled transcription units do. Similarly, close to 40% of the B. subtilis σ^E^-controlled transcription units are present in *Clostridium,* but only about 20% of the σ^K^-controlled transcription units are present. An appealing explanation for the lower level of conservation among σ^K^ regulons is that genes switched on late in the mother-cell line of gene expression are enriched for genes encoding components of the outer surface of the spore—proteins that are likely to undergo the greatest evolutionary adaptation to the ecological niche in which a particular species is found. Indeed, experiments involving the use of atomic force microscopy reveal that the surfaces of the spores of the closely related species *B. subtilis, B. anthracis,* and B. cereus exhibit quite distinctive landscapes (Chada et al. 2003).

### Conclusions

We have provided a comprehensive description of the program of gene transcription for a single differentiating cell type and have shown that this program is governed by a regulatory circuit involving the action of five transcriptional control proteins acting as activators or repressors or both. The underlying logic of the circuit is that of a linked series of coherent and incoherent type-1 FFLs involving two-way combinations of the five regulatory proteins. The circuit is expected to create pulses of gene transcription in which large numbers of genes are switched on and subsequently switched off. We anticipate that type-1 FFLs linked in series are likely to be a common feature of programs of cellular differentiation in a wide variety of developing systems.

## Materials and Methods

### 

#### Strains

All strains used here are derivatives of the wild-type strain PY79, with the exception of the σ^K^ overproducing strain, which is a derivative of strain 168. Strains PE436 and PE437 (Eichenberger et al. 2003), PE452, PE454, PE455, PE456, and SW282 were used for transcriptional profiling under conditions of sporulation. PE452 was obtained by transformation of strain RL560 to MLS resistance with chromosomal DNA from strain MO1027 (*spoIVCB*::*erm,* a gift from P. Stragier, Institut de Biologie Physico-Chimique, Paris) (*sigG*::*cat;* a derivative in the PY79 background of strain MO479.2 [Karmazyn-Campelli et al. 1989]). PE454 was generated by transformation of PY79 to chloramphenicol resistance with chromosomal DNA from strain RL16 (*gerE*::*cat;* Cutting and Mandelstam 1986). PE455 is the result of the transformation of strain PE454 with chromosomal DNA from strain MO1027. PE456 was obtained by transformation of PE452 to spectinomycin resistance with chromosomal DNA from strain PE239 (*spoIIIDΔ*::*spc;* Eichenberger et al. 2001). SW282 was generated by transformation of PE454 to spectinomycin resistance with chromosomal DNA from strain PE316 (*ylbOΔ*::*spc;* Eichenberger et al. 2003). Strain SI01, which was used for overproduction of σ^K^, was created by double cross-over recombination at the *amyE* locus, following transformation with XhoI-digested plasmid pMFNsigK. Strains PE511, PE551, PE553, PE558, PE568, and SW312 were used for β-galactosidase activity assays. PE511 is a derivative in the PY79 background of strain MO1533 (*amyE*::*spoIIP–lacZ cat;* Frandsen and Stragier 1995). PE551 and PE553 were obtained by double cross-over recombination of XhoI-digested plasmids pPE72 and pPE74, respectively, into PY79 and selection for chloramphenicol resistance and spectinomycin sensitivity. SW312, PE568, and PE558 were generated by transformation with chromosomal DNA from strain PE316 to spectinomycin resistance of strains PE551, PE511, and PE553, respectively. Strains PE529 *(yheDΔ*::*erm),* PE534 *(mprΔ*::*tet),* PE538 *(yqfTΔ*::*kan),* PE548 *(lipΔ*::*erm),* PE549 *(cotTΔ*::*spc),* RL3231 *(ycgMΔ*::*tet),* PE566 *(cotVWΔ*::*erm),* PE569 *(ydcIΔ*::*tet),* and PE570 *(yheIΔ*::*spc)* were generated with the technique of long-flanking homology PCR (Wach 1996). The sequence of the primers used for gene inactivation is available upon request. Strain PE563 *(cotFΔ*::*cat)* was obtained by transformation of PY79 to chloramphenicol resistance with chromosomal DNA from RL654 (Cutting et al. 1991c). PE564 is the equivalent of strain MO1057 (*spoIVCA*::*erm;* a gift from P. Stragier) in the PY79 background. Strains RL2391, RL2396, and RL2519 are from Eichenberger et al. 2001 and RL2393, RL2559, RL2570, RL2571, and RL2581 are from Eichenberger et al. 2003. Strain BDR107 is a derivative of PY79 harboring *spoIVCB*::*erm* from strain MO1027. BDR362 corresponds to RL75 (*spoIIID*::*erm;* Kunkel et al. 1989). P*_spoIVF_pro*–*sigK spc* from pDR191 was introduced into the *amyE* locus of BDR107 to generate BDR1663. Genomic DNA from BDR1663 was used to transform BDR362 to spectinomycin resistance to generate BDR1666.

#### Plasmids

Plasmid pMFN20 was constructed by cloning a PstI (blunted)-EcoRI (blunted) 1.3-kb Neo^r^ cassette from pBEST501 (Itaya et al. 1989) and a 7.2-kb DNA fragment from plasmid pMF20 (M. F., unpublished data), amplified with primers SI1 (5′-ATGGATGAGCGATGATGATATCCGT-3′) and SI2 (5′-AACTATTGCCGATGATAAGCTGTC-3′). The pro-less *sigK* gene was constructed using recombinant PCR (Wach 1996). A DNA fragment containing the upstream region of the *sigK* gene was amplified from chromosomal DNA of strain 168 using primers SI3 (5′-CCCAAGCTTTTAGTATGCTGCTTACC-3′) and SI4 (5′-AAG*GCATTGTTTTTCACGTA*CATCGTCACCTCCACAAAAGTAT-3′) (restriction site is underlined; the sequence complementary to primer SI5 is italicized). The other primer set, primer SI5 (5′-TACGTGAAAAACAATGC-3′) and primer SI6 (5′-CGCGGATCCTTCTGCATTATTTCCCC-3′), was used for PCR amplification of chromosomal DNA of strain 168 isolated from cells 6 h after initiation of sporulation to generate the rearranged *sigK* gene. The two PCR products were fused by PCR to generate the pro-less *sigK* gene. Plasmid pMFNsigK was constructed by cloning the pro-less *sigK* fragment into pMFN20 between HindIII and BamHI.

Plasmids pPE72 *(amyE*::P*_spoIIM_–lacZ cat)* and pPE74 *(amyE*::P*_yqhV_–lacZ cat)* were obtained as follows. Primers PE857 (5′-CGTCTAGCCGAATTCCAGCACACCATCTTTCAGCACACA-3′) and PE858 (5′-CGTATCCCGGGATCCCGCCCGCTCTAGTGATTTGATTTA-3′) were used to amplify the promoter sequence of *spoIIM* from PY79 chromosomal DNA by PCR (restriction sites are underlined), whereas primers PE860 (5′-CGTCTAGCCGAATTCGGCTTGTTGTAAACGTGCCGTTCT-3′) and PE861 (5′-CGTATCCCGGGATCCCCCATTTTTTTATATGATATGCTCT-3′) were used for the PCR amplification of the promoter sequence of *yqhV.* The PCR products were gel-purified (QIAquick Gel extraction kit; Qiagen, Valencia, California, United States) and digested with EcoRI and BamHI. In parallel, vector pDG1661 (Guerout-Fleury et al. 1996) was similarly digested with EcoRI and BamHI and treated with shrimp alkaline phosphatase (USB). The digested vector and PCR fragments were purified (QIAquick PCR purification kit; Qiagen) and ligated using T4 DNA ligase. Ligations were transformed in DH5α cells to ampicillin resistance, and plasmids pPE72 and pPE74 were recovered by alkaline lysis.

Primers odr261 (5′-GGCAAGCTTGTGGAGGTGACGATGGTGACAG-3′) and odr262 (5′-GGCGGATCCTGCGGGAGGATTATAAGTCAAG-3′) were used to amplify *pro–sigK* from pSK6 (Kunkel et al. 1990). The PCR product was cloned into pdr77 (Rudner and Losick 2002) between HindIII and BamHI to generate pdr191 *(amyE*::P*_spoIVF_*–*pro*–*sigK spc).*

#### Growth and sporulation conditions

Strains used for transcriptional profiling, β-galactosidase activity assays, and ChIP-on-chip experiments were grown in hydrolyzed casein medium at 37 °C to an A_600 nm_ of 0.6. Pellets obtained by centrifugation were suspended in Sterlini–Mandelstam medium (Sterlini and Mandelstam 1969; Harwood and Cutting 1990) and placed in a shaking water bath at 37 °C. Samples were collected at the indicated times after resuspension.

A fresh colony of the σ^K^ overproducing strain SI01 was grown in 5 ml of Penassay broth (Difco Laboratories, Detroit, Michigan, United States) overnight at 30 °C. Next, 50 ml of LB broth with and without 10 mM of xylose was inoculated with 1 ml of the overnight culture. Cells were grown by incubation at 37 °C with shaking and harvested 2 h after induction (A_600 nm_ of 0.8) for extraction of RNA.

#### Transcriptional profiling

DNA microarrays were generated as described by Britton et al. (2002). RNA preparation, sample labeling, and hybridization procedures were performed as described by Eichenberger et al. (2003). Expression data were obtained from three independent experiments for SpoIIID, GerR, σ^K^, and GerE. Our statistical analysis procedure, described in detail by Conlon et al. (2004), was performed separately for each set of microarrays for SpoIIID, GerR, σ^K^, and GerE. Normalization of each slide was performed using an iterative rank-invariant method. A Bayesian hierarchical model incorporating experimental variation was used to combine normalized slides across replicated experiments. A Markov chain Monte Carlo implementation of the model with 4,000 iterations produced a posterior median estimate of the log-expression ratio for each gene, and the corresponding Bayesian confidence interval. Genes were scored for the posterior probability of a positive log-expression ratio. Genes with scores above or equal to a threshold of 0.95 were determined to be upregulated in an experimental condition, and genes with scores below or equal to a threshold of 0.05 to be downregulated. Finally, genes in the upregulated category with a nonlogarithmic expression ratio inferior to a threshold of 2.0 and, similarly, genes in the downregulated category with an expression ratio superior to a threshold of 0.5 were not included, unless indicated otherwise (Tables S2 and S4) in the list of differentially expressed genes. The data are available online in MIAME-compliant format at http://mcb.harvard.edu/losick and were also deposited in the Gene Expression Omnibus database under the accession number GSE1620.

#### Overexpression and purification of SpoIIID protein

SpoIIID protein was overproduced by the T7 promoter overexpression system of *Escherichia coli.* The SpoIIID protein expression plasmid was constructed by amplifying the corresponding region from PY79 chromosomal DNA using primers 5′-TACATATGCACGATTACATCAAAGAG-3′ and 5′-CCCTCGAGCGATTGCTGAACAGGCTC-3′. The PCR fragment was digested by NdeI and AvaI and ligated into the NdeI/AvaI-digested vector pET22b (Novagen, Madison, Wisconsin, United States) to generate the SpoIIID protein expression plasmid (pETIIID). The plasmid was transformed into strain BL21 (DE3). Cells carrying pETIIID were grown at 37 °C in 2 l of LB containing 100 μg/ml of ampicillin to an A_600 nm_ of 0.6, at which point T7 RNA polymerase synthesis was induced by the addition of IPTG to a final concentration of 1 mM. Cells were harvested 5 h later by centrifugation. The pellet was resuspended in 40 ml of binding buffer (50 mM Tris-HCl [pH 8.0], 500 mM NaCl, 5 mM imidazole) and disrupted by sonication. Cell debris was removed by centrifugation at 20,000*g* for 30 min and the supernatant was loaded on 1 ml of Ni^2+^–NTA agarose resin (Qiagen) equilibrated with binding buffer. SpoIIID was eluted with a 20-ml imidazole gradient from 5 to 500 mM in binding buffer. Peak fractions were pooled and dialyzed against TGED buffer. The amount of protein was determined with a Bio-Rad (Hercules, California, United States) protein determination kit with BSA as the standard. The purified SpoIIID protein was tested in an in vitro transcription system using reconstituted σ^E^–RNA polymerase and *spoIID* as template (data not shown).

#### Gel EMSAs

DNA fragments of interest were obtained by PCR amplification of PY79 chromosomal DNA (see Supporting Information for the description of primers used), gel-purified (QIAquick Gel extraction kit), and end-labeled with T4 polynucleotide kinase in the presence of [γ–^32^P]-ATP for 30 min at 37 °C. After labeling, the fragments were purified with the QIAquick PCR purification kit. Prior to loading on a 6% polyacrylamide–0.16% Bis-0.5X TBE gel that had been prerun at 200 V for 1 h, DNA fragments were preincubated in gel shift-binding buffer (10 mM Tris-HCl [pH 7.5], 50 mM NaCl, 1 mM EDTA, 5% glycerol, 1 mM DTT, and 50 μg/ml BSA) for 30 min at room temperature with various amounts of purified SpoIIID protein (0 nM, 50 nM, 100 nM, and 200 nM). The gel was run for 1 h at 200 V, dried, and exposed to Kodak X-OMAT film (Kodak, Rochester, New York, United States).

The following DNA fragments were used in the analysis: *abrB, spoIID, spoIIG,* and *racA* (Molle et al. 2003a), *bofA* (from nucleotide 29439 to 30030), *spoIVCA* (from 2654348 to 2654008), *asnO* (from 1156248 to 1156617), *cotE* (from 1774088 to 1774414), *cotF* (from 4165851 to 4166189), *cotT* (from 1280431 to 1280108), *gerM* (from 2902355 to 2902020), *spoIVA* (from 2387164 to 2386826), *spoIVFA* (from 2856840 to 2856657), *spoVB* (from 2828500 to 2828872), *spoVK* (from 1873045 to 1873470), *yabP* (from 67986 to 68297), *ybaN* (from 160708 to 160468), *ycgF* (from 333921 to 334278), *yitE* (from 1174319 to 1174179), *ykvU* (from 1448417 to 1448728), *ylbJ* (from1571288 to 1570927), *ypjB* (from 2361797 to 2361463), *yqfC* (from 2616344 to 2616016), *yqfZ* (from 2587752 to 2588021), *albE–albF*1 (from 3838793 to 3839220), *albE–albF*2 (from 3839214 to 3839656), *albE–albF*3 (from 3839637 to 3840082), *albE–albF*4 (from 3840067 to 3840481), *dctR–dctP*1 (from 498913 to 499280), *dctR–dctP*2 (from 499263 to 499554), *dctR–dctP*3 (from 499531 to 499913), *dctR–dctP*4 (from 499912 to 500356), *tenI–goxB–thiS*1 (from 1242878 to 1243331), *tenI–goxB–thiS*2 (from 1243322 to 1243755), *tenI–goxB–thiS*3 (from 1243749 to 1244115), *tenI–goxB–thiS*4 (from 1244063 to 1244366), *treA–treR–yfkO*1 (from 852742 to 853109), *treA–treR–yfkO*2 (from 853071 to 853409), *treA–treR–yfkO*3 (from 853387 to 853810), *treA–treR–yfkO*4 (from 853797 to 854209), *yfmC–yfmD*1 (from 825948 to 825585), *yfmC–yfmD*2 (from 825607 to 825255), *yfmC–yfmD*3 (from 825266 to 824884), *yfmC–yfmD*4 (from 824903 to 824489).

#### DNAase I footprinting

DNAase I footprinting was carried out as described by Fujita and Sadaie (1998).

#### Antibodies for SpoIIID

The C-terminus of the SpoIIID protein was overproduced by the T7 promoter overexpression system of E. coli and used as an antigen for the production of anti-SpoIIID antibodies. The SpoIIID C-terminus protein expression plasmid was constructed by amplifying the region from PY79 chromosomal DNA using primers, 5′-GAAGCTAGCATGATTAACCCCGACTTGGCAAACG-3′ and 5′-GAACTCGAGCGATTGCTGAACAGGCTCTCCTT-3′. The PCR fragment was digested by NheI and XhoI and ligated into the NheI/XhoI-digested vector pET21b (Novagen) to generate the SpoIIID C-terminus protein expression plasmid pMF213. Overexpression and purification of the protein are described in a previous section. The anti-SpoIIID antibodies were prepared by Covance Research Products (Denver, Pennsylvania, United States) and were highly specific as judged by Western blot analysis, which revealed only a single cross-reacting species.

#### Chromatin immunoprecipitation in combination with gene microarrays (ChIP-on-chip)

Three hours after resuspension of PY79 cells in Sterlini-Mandelstam medium at 37 °C, cross-links were generated by treatment with formaldehyde (1% final concentration) for 30 min. The rest of the procedure was identical to the one described by (Molle et al. 2003a, 2003b).

The data analysis for the ChIP-on-chip experiments was carried out using the Resolver statistical package (Rosetta, Seattle, Washington, United States). Experiments were normalized and combined for enrichment-factor determination (Rosetta Resolver). An enrichment factor for a given gene represents the ratio of immunoprecipitated DNA to total DNA. It was considered significant when higher than 2 and with an associated *p*-value lower than 0.001.

#### BioProspector/BioOptimizer

BioProspector (Liu et al. 2001) is a stochastic motif-discovery program used to find conserved subsequences of fixed width in a set of DNA sequences, based on a statistical motif-discovery model reviewed in Jensen et al. (2004). The program can also be used for motifs consisting of two conserved blocks connected with a variable-length gap of unconserved nucleotides, and BioProspector can also be forced to find sites in every input sequence. Since BioProspector is a stochastic algorithm, more than one possible motif can be found, and since the program requires the motif width to be fixed, several different fixed widths should be used in the usual case where the motif width is not known. Thus, we collected the top five Bioprospector motifs under a range (6–12 bps) of seven fixed widths, giving a total of 35 putative motifs.

BioOptimizer (Jensen and Liu 2004) is an optimization program designed to improve the results of each discovered BioProspector motif and to score each motif so that the “best” putative motif can be selected out of the 35 we discovered. The scoring function used is the exact log-posterior density of the Bayesian motif-discovery model given in Jensen and Liu (2004). Starting from the set of sites predicted by BioProspector, the scoring function is optimized by accepting the addition of new motif sites or removal of current motif sites only if these changes increase the score. BioOptimizer also has the flexibility to allow the motif width to vary, so that the “best” width can also be determined. As well, BioOptimizer can be restricted to force particular sequences in the dataset to contain at least one site while leaving other sequences unrestricted. This property was utilized in our SpoIIID motif search, where a subset of sequences has additional biochemical evidence that they contain at least one SpoIIID-binding site.

Having found an optimal motif with our combined BioProspector/BioOptimizer procedure, we implemented an additional scanning procedure to find more potential SpoIIID sites. Using the estimated proportion of nucleotide *k* in position *j* of the motif (θ^_*j,k*_ ) and the estimated proportion of nucleotide *k* in the background (θ^_*j,k*_ ) provided by our optimal motif, we scanned all upstream sequences to see if there were additional sites that matched our discovered motif closely but were not strong enough to be detected by the motif-discovery procedure. In each sequence, for each potential starting position *i,* we had a potential site *S*_*i*_ = *r_i_*, *r*_*i*+l_, …, *r*_*i*+*w*−l_) , for which we compute the following score:



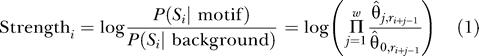



We considered the site in each sequence with the largest Strength value to be the best candidate as an additional site. If a sequence already contained a site found by our motif-discovery procedure, we would expect that this same site would be the one with the largest Strength value. For any sequence that did not have an optimal site found by the motif-discovery procedure, this scanning procedure gave us new site predictions. However, for any new sites found by the scanning procedure, one must be cautious about the strength of these sites, since the procedure found sites in each sequence regardless of how well those sites matched our optimal motif. Therefore, we also calculated a *p*-value for each site by comparing the Strength value calculated for that site to the Strength value calculated for 10,000 random sequences. Only sites with low *p*-values were considered as potential sites. With the Bonferroni correction for multiple comparisons, we considered only sites with *p*-values less than 0.000183.

#### MDscan analysis of the SpoIIID-binding motif

We used the word-enumeration algorithm “MDscan” (Liu et al. 2002) to identify motifs in sequences most enriched by immunoprecipitation experiments. In this algorithm, it is assumed that the most enriched sequences have stronger motif signals than the remaining sequences. MDscan first identifies oligomers of width *w* (*w*-mers) in the top sequences, which are used as seed oligomers. Motif matrices are constructed for each seed oligomer using all similar segments from the top sequences. Segments are defined to be similar if they share at least *m* matched positions, with *m* determined so that the probability that a pair of randomly produced *w*-mers are *m*-matches is less than 0.15%. The resulting motif matrices are evaluated using the following semi-Bayesian scoring function:



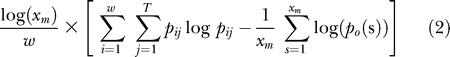



where *x_m_* is the number of segments aligned in the motif, *p_ij_* is the frequency of base *j* at motif position *i,* and *p_o_(s)* is the probability of generating segment *s* from the background model. The top distinct highest scoring motifs are defined as candidate motifs. These motifs are refined using the remaining sequences, by adding new *w*-mers to the matrix if the score is increased. The motifs are further refined by reexamining all segments of the motif matrix and removing segments if the motif score is increased.

We first ranked by enrichment ratio the 26 regions of the chromosome that were enriched by immunoprecipitation by a factor of 2 or greater. We used the top 20 regions as the top sequences, with the remaining six sequences used for refinement. The 26 regions were used as the background sequences, and we reported 30 candidate motifs. We first searched for motifs of width *w* = 8. In using alternative widths (*w* = 7, 9, and 10), and alternative definitions of top regions (15–25), the top reported motif was similar to that for width 8. As reported in Liu et al. (2002), MDscan is tolerant of different top sequence definitions (∼3–20), and of moderate ranking errors.

#### Germination assays

Tests for germination using 2,3,5-triphenyltetrazolium chloride overlay were carried out as described in Nicholson and Setlow (1990). Strains mutant for GerR (PE316) were compared to wild-type cells (PY79), and strains mutant for GerE (strain PE454) or CotE (strain RL322; Driks et al. 1994) were described as negative controls. All strains were sporulated in DSM. Heat activation was performed in a 65 °C oven for 3 h.

#### Measuring β-galactosidase activity

β-galactosidase activity assays were carried out as previously described (Miller 1972; Harwood and Cutting 1990).

#### Promoter mapping by 5′ RACE–PCR

The 5′ end of several σ^K^-controlled mRNAs was determined by the RACE–PCR procedure (Frohman 1994; Price et al. 2001). Total RNA was extracted from strains PE454 *(sigE* ^+^ *, sigK* ^+^ *)* and PE455 *(sigE* ^+^ *, sigK* ^−^ *)* and analyzed as described by Eichenberger et al. (2003).

#### Measuring sporulation efficiency

Strains were grown to exhaustion in DSM for 30 h at 37 °C and assayed for heat resistance as previously described by van Ooij et al. (2004).

## Supporting Information

Figure S1 DNAase I Footprinting of SpoIIID Binding to the Promoters of *spoIID, spoIIIA,* and *spoVE*
(A) Radioactive DNA fragments were incubated with no protein (left lane) or with 400 nM of SpoIIID protein (right lane) and then subjected to DNAaseI footprinting. A chemical sequencing ladder was used as a marker (not shown). Protected regions are indicated by a bar.
(B) Position of SpoIIID-binding sites. The nucleotide sequence upstream of the transcriptional start site (+1) is shown for *spoIID, spoIIIA, spoVE*-P1, and *spoVE*-P2. The boundaries of the region protected from DNAase I digestion by SpoIIID are indicated by bars. The bold letters identify the sequences within the protected regions that match with the SpoIIID consensus sequence.
(1.72 MB PPT).

Figure S2 Mapping of Transcription Start Sites by 5′ RACE–PCR The underlined uppercase bold letters identify the 5′ ends of mRNAs from σ^K^-controlled genes as determined by RACE–PCR. Also indicated are the corresponding −35 and −10 regions (uppercase letters in bold), the ribosome-binding site (double underlining), and the translation start site (uppercase letters). RNA collected from strain PE454 *(sigE* ^+^ *sigK* ^+^ *)* and strain PE455 *(sigE* ^+^, *sigK* ^−^ *)* was used for the determination of transcription start sites. In four cases indicated with an asterisk *(yfnE, yhcO, yitC,* and *ypqA),* an identical transcription start site was identified for strains PE454 and PE455, which is interpreted as evidence that the promoters for these three transcription units are recognized both by σ^E^ and σ^K^. In all of the other cases, a transcription start site was obtained only with RNA collected from strain PE454.
(22 KB DOC).

Table S1 Mother-Cell Gene Expression (1.5 MB XLS).

Table S2 Effect of SpoIIID and GerR on the expression of genes in the σ^E^ regulon (351 KB XLS).

Table S3 ChIP-on-chip data for SpoIIID (332 KB XLS).

Table S4 Effect of GerE on the expression of genes in the σ^K^ regulon (238 KB XLS).

### Accession Numbers

The Swiss-Prot (http://www.ebi.ac.uk/swissprot/) accession numbers for the gene products discussed in this paper are σ^E^ (P06222), σ^G^ (P19940), σ^K^ (P12254), BofA (P24282), CodY (P39779), GerE (P11470), GerR (O34549), RacA (P45870), Spo0A (P06534), SpoIIID (P15281), SpoIVFA (P26936), and SpoIVFB (P26937).

The GenBank (http://www.ncbi.nlm.nih.gov/Genbank) accession numbers for the genes discussed in this paper are *abrB* (BSU00370), *albA* (BSU37370), *albB* (BSU37380), *albC* (BSU37390), *albD* (BSU37400), *albE* (BSU37410), *albF* (BSU37420), *argC* (BSU11190), *argJ* (BSU11200), *asnO* (BSU10790), *azlB* (BSU26720), *azlC* (BSU26710), *azlD* (BSU26700), *bnrQ* (BSU26690), *bofA* (BSU00230), *cotA* (BSU06300), *cotD* (BSU22200), *cotE* (BSU17030), *cotF* (BSU40530), *cotH* (BSU36060), *cotM* (BSU17970), *cotT* (BSU12090), *cotV* (BSU11780), *cotW* (BSU11770), *ctpB/yvjB* (BSU35240), *cwlC* (BSU17410), *cwlH* (BSU25710), *cwlJ* (BSU02600), *cypA* (BSU26740), *cysC* (BSU15600), *cysH* (BSU15570), *cysK* (BSU00730), *cysP* (BSU15580), *dctP* (BSU04470), *dctR* (BSU04460), *exuR* (BSU12370), *exuT* (BSU12360), *gerE* (BSU28410), *gerM* (BSU28380), *gerPA* (BSU10720), *gltR* (BSU26670), *goxB* (BSU11670), *kapD* (BSU31470), *lip* (BSU31470), *mpr* (BSU02240), *phoB* (BSU05740), *proH* (BSU18480), *proJ* (BSU18470), *racA/ywkC* (BSU37030), *safA* (BSU27840), *sat* (BSU15590), *spoIID* (BSU36750), *spoIIGA* (BSU15310), *spoIIIAA* (BSU24430), *spoIIIAB* (BSU24420), *spoIIIAF* (BSU24380), *spoIIM* (BSU23530), *spoIIP* (BSU25530), *spoIVA* (BSU22800), *spoIVCA* (BSU25770), *spoIVCB* (BSU25760), *spoIVFA* (BSU27980), *spoIVFB* (BSU27970), *spoVD* (BSU15170), *spoVE* (BSU15210), spoVID (BSU28110), *spoVK* (BSU17420), *spoVM* (BSU15810), *spsA* (BSU37910), *spsG* (BSU37850), *spsJ* (BSU37830), *tenI* (BSU11660), *thiS* (BSU11680), *treA* (BSU07810), *treR* (BSU07820), *uxuA* (BSU12340), *ybaN* (BSU01570), *ybaS* (BSU01590), *ycgF* (BSU03090), *ycgM* (BSU03200), *ycgN* (BSU03210), *ydcI* (BSU04780), *ydhF* (BSU05730), *yeeA* (BSU06760), *yeeB* (BSU06770), *yeeC* (BSU06780), *yefA* (BSU06730), *yefB* (BSU06740), *yefC* (BSU06750), *yfhP* (BSU08620), *yfkO* (BSU07830), *yfmC* (BSU07520), *yfmD* (BSU07510), *yfnD* (BSU07310), *yfnE* (BSU07300), *yfnF* (BSU07290), *yfnG* (BSU07280), *yfnH* (BSU07270), *yhbB* (BSU08920), *yhbH* (BSU08980), *yhcO* (BSU09160), *yhcP* (BSU09170), *yheC* (BSU09780), *yheD* (BSU09770), *yheH* (BSU09720), *yheI* (BSU09710), *yhjL* (BSU10550), *yisZ* (BSU10910), *yitA* (BSU10920), *yitB* (BSU10930), *yitC* (BSU10940), *yitD* (BSU10950), *yitE* (BSU10960), *yjcA* (BSU11790), *yjcM* (BSU11910), *yjcN* (BSU11920), *yjcO* (BSU11930), *yjmC* (BSU12320), *yjmD* (BSU12330), *yjmF* (BSU12350), *yknT* (BSU14250), *yknU* (BSU14320), *yknV* (BSU14330), *ykuD* (BSU14040), *ykvI* (BSU13710), *ykvU* (BSU13830), *ylbJ* (BSU15030), *ylbO/gerR* (BSU15090), *yngE* (BSU18210), *yngF* (BSU18220), *yngG* (BSU18230), *yngH* (BSU18240), *yngI* (BSU18250), *yngJ* (BSU18260), *yoaB* (BSU18540), *yoaD* (BSU18560), *yodU* (BSU19810), *ypqA* (BSU22240), *ypqP* (BSU21670), *yqfT* (BSU25120), *yqhV* (BSU24440), *yrdK* (BSU26680), *yydB* (BSU40220), *yydC* (BSU40210), *yydD* (BSU40200), *yydG* (BSU40170), *yydH* (BSU40160), *yydI* (BSU40150), and *yydJ* (BSU40140).

Microarray data were deposited in the Gene Expression Omnibus database under the accession number GSE1620, where they are accessible at http://www.ncbi.nlm.nih.gov/geo/query/acc.cgi?acc=GSE1620.

## Abbreviations

ChIP-on-chip: chromatin-immunoprecipitation in combination with gene microarrays
EMSA: electrophoretic mobility-shift assay
FFL: feed-forward loop
MDscan: Motif Discovery scan
RACE–PCR: rapid amplification of complementary DNA ends–PCR
