# Supplementary material for: The Program of Gene Transcription for a Single Differentiating Cell Type during Sporulation in Bacillus subtilis
Source: PLoS Biol. 2004 Sep 21;2(10):e328. doi: 10.1371/journal.pbio.0020328 (PMC517825; doi:10.1371/journal.pbio.0020328)
Supplement: Figure S1 — (A) Radioactive DNA fragments were incubated with no protein (left lane) or with 400 nM of SpoIIID protein (right lane) and then subjected to DNAaseI footprinting. A chemical sequencing ladder was used as a marker (not shown). Protected regions are indicated by a bar. (B) Position of SpoIIID-binding sites. The nucleotide sequence upstream of the transcriptional start site (+1) is shown for spoIID, spoIIIA, spoVE-P1, and spoVE-P2. The boundaries of the region protected from DNAase I digestion by SpoIIID are indicated by bars. The bold letters identify the sequences within the protected regions that match with the SpoIIID consensus sequence. (1.72 MB PPT). [file pbio.0020328.sg001.ppt]

## Slide 1
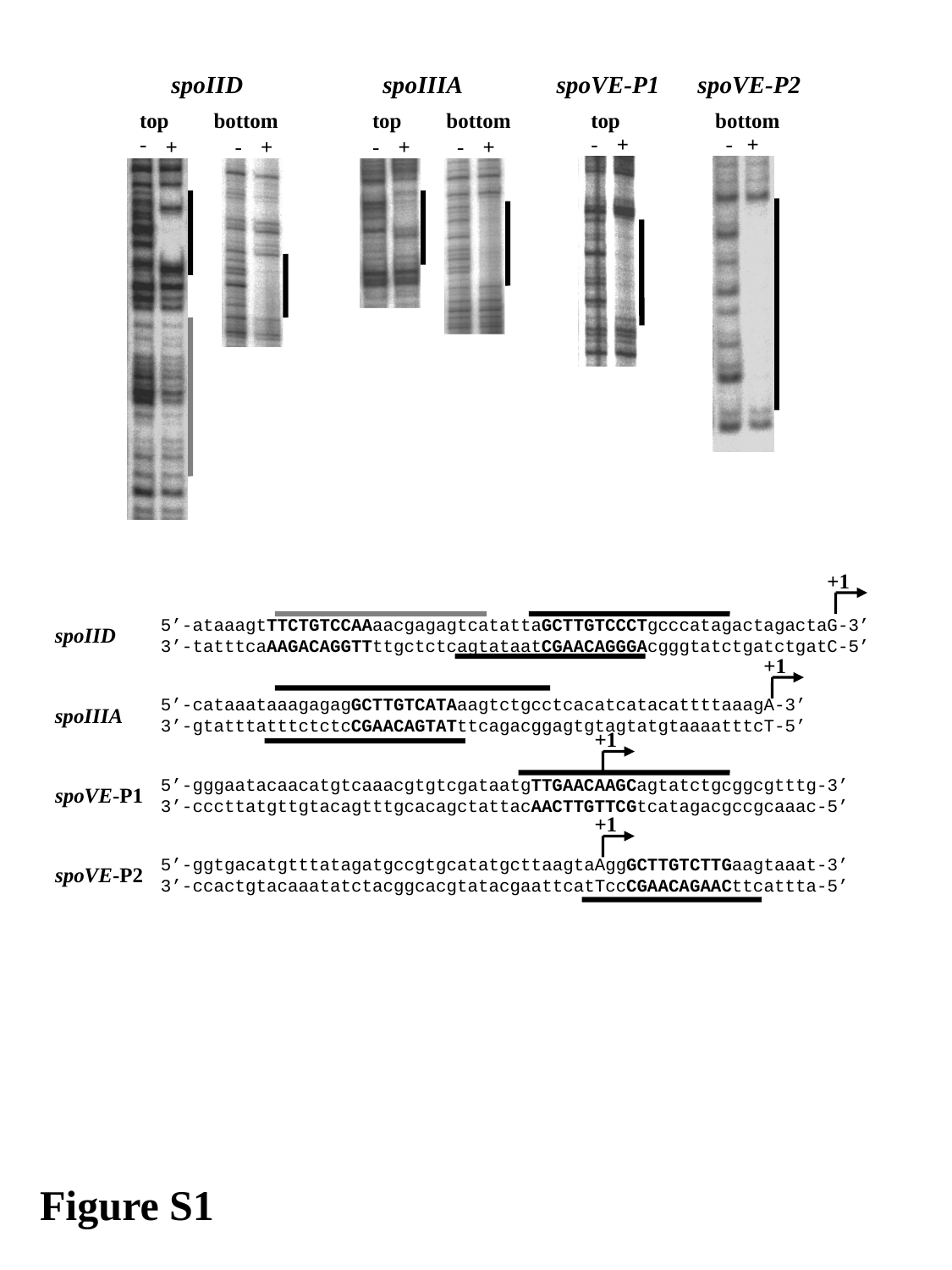

spoIID
spoIIIA
spoVE-P1
spoVE-P2
top
bottom
top
bottom
top
bottom
-
-
+
-
+
+
-
+
-
+
-
+
+1
5’-ataaagtTTCTGTCCAAaacgagagtcatattaGCTTGTCCCTgcccatagactagactaG-3’
3’-tatttcaAAGACAGGTTttgctctcagtataatCGAACAGGGAcgggtatctgatctgatC-5’
spoIID
+1
5’-cataaataaagagagGCTTGTCATAaagtctgcctcacatcatacattttaaagA-3’
3’-gtatttatttctctcCGAACAGTATttcagacggagtgtagtatgtaaaatttcT-5’
spoIIIA
+1
5’-gggaatacaacatgtcaaacgtgtcgataatgTTGAACAAGCagtatctgcggcgtttg-3’
3’-cccttatgttgtacagtttgcacagctattacAACTTGTTCGtcatagacgccgcaaac-5’
spoVE-P1
+1
5’-ggtgacatgtttatagatgccgtgcatatgcttaagtaAggGCTTGTCTTGaagtaaat-3’
3’-ccactgtacaaatatctacggcacgtatacgaattcatTccCGAACAGAACttcattta-5’
spoVE-P2
Figure S1
