# Supplementary material for: The Program of Gene Transcription for a Single Differentiating Cell Type during Sporulation in Bacillus subtilis
Source: PLoS Biol. 2004 Sep 21;2(10):e328. doi: 10.1371/journal.pbio.0020328 (PMC517825; doi:10.1371/journal.pbio.0020328)
Supplement: Figure S2 — The underlined uppercase bold letters identify the 5′ ends of mRNAs from σK-controlled genes as determined by RACE–PCR. Also indicated are the corresponding −35 and −10 regions (uppercase letters in bold), the ribosome-binding site (double underlining), and the translation start site (uppercase letters). RNA collected from strain PE454 (sigE + sigK + ) and strain PE455 (sigE +, sigK − ) was used for the determination of transcription start sites. In four cases indicated with an asterisk (yfnE, yhcO, yitC, and ypqA), an identical transcription start site was identified for strains PE454 and PE455, which is interpreted as evidence that the promoters for these three transcription units are recognized both by σE and σK. In all of the other cases, a transcription start site was obtained only with RNA collected from strain PE454. (22 KB DOC). [file pbio.0020328.sg002.doc]

**Figure S2. Mapping of transcription start sites by 5’-RACE-PCR**

*oxdD* (*yoaN*) ctag**gcaca**gcaatctaagattctg**cataggct**gaaat**aaaat**cttgttcatttctaaaacgaggtgcatgctgTTG

*spsA* ccta**gcgca**acttgagcataagcaa**cataagat**aacgat**ag**ttt-15 bp-aagaaggaggtgtaggatTTG

*tgl* taac**tttca**ttgcccaagctctttg**catatctt**at**aaaaaa**caaggggggctaaacATG

*ylbD* aggc**gaaca**caatgcccgacaaaac**gatacatt**gtagt**ag**gtaacgattttcggagggggtgaggattGTG

*yfnE** gtga**aagca**agcgttattattcctg**catataat**tcga**ag**gagc-75 bp-ctctgaggacgggacgctctcgatg

*yfnH* ttgg**tcacc**aaggctggctttctct**catatcat**tacagt**ag**attt-85 bp-atagagatgaggggtagagATG

*yhcO** aatg**acaca**ctgcgaactcaggctg**catagagt**aaaaat**aaaaag**gtaca-70 bp-tgtgcggagtccttgtgATG

*yhjR* ctcc**gcaca**tctctcc-ctgcccaa**catatact**tttac**ag**aagcccaattcctaagaatggagttgaatccccTTG

*yitB* acct**gaacc**tttatgc-aaaaaatg**aatagtct**gcctat**aac**tcga-20 bp-atgcaaacggggggaaaacaTTG

*yitC** tgcg**gaacc**attaagagcccggctg**aatatgct**ttttagc**aaaat**ggttttatgcccaaggagaggtagtATG

*ykuD* aatg**tagca**ggatgggctcctcatc**aatactta**taagt**aaa**aaaggaggatgaggaaaCTG

*yngK* ttaa**tcacc**cttttat-agcccggg**aataccgt**aatagc**g**aata-15 bp-tgggggagaagaaacagaATG

*yodH* tcat**aaacc**tatgtct-ttatacga**catatgat**aagg**aa**aaaggaggattcgataTTG

*ypqA** ttta**taaca**acatctggcatagacg**cataatct**ggtt**aaa**aaaggcggtggcgataTTG

*yrkC* ttta**gcaca**ctccctt-aataaatg**cataggat**actatc**ac**aaaaatttataattgcggggatcaaaaaATG

*ysnD* atag**gcact**tctcttgttccctcgg**catacatt**aatg**atat**cttgcaaagaaaaggtgatacgATG

*ytlA* gcat**tcaca**cattttt-atacccat**catacgat**atgt**aaag**caaa-20 bp-gaaataaggaggttgtctatGTG

*yxeE* atgc**gtgcc**actgtgccaatgacta**cataagtt**at**aag**gaattcacccacaaggagactgatcacATG
